# Testing for Network Specificity in Brain‐Behavior Associations Using Ordinal Dominance Curves

**DOI:** 10.1002/hbm.70493

**Published:** 2026-03-23

**Authors:** Noah Hillman, Sarah M. Weinstein, Joëlle Bagautdinova, Kevin Y. Sun, Matthew Cieslak, Taylor Salo, Yong Fan, Arielle S. Keller, Aaron F. Alexander‐Bloch, Simon N. Vandekar, Armin Raznahan, Theodore D. Satterthwaite, Haochang Shou, Russell T. Shinohara

**Affiliations:** ^1^ Penn Statistics in Imaging and Visualization Endeavor, Department of Biostatistics, Epidemiology, and Informatics, Perelman School of Medicine University of Pennsylvania Philadelphia Pennsylvania USA; ^2^ Department of Epidemiology and Biostatistics Temple University College of Public Health Philadelphia Pennsylvania USA; ^3^ Penn Lifespan Informatics and Neuroimaging Center, Department of Psychiatry, Perelman School of Medicine University of Pennsylvania Philadelphia Pennsylvania USA; ^4^ Lifespan Brain Institute (LiBI) Children’s Hospital of Pennsylvania and Perelman School of Medicine Philadelphia Pennsylvania USA; ^5^ Department of Psychiatry, Perelman School of Medicine University of Pennsylvania Philadelphia Pennsylvania USA; ^6^ Center for AI and Data Science for Integrated Diagnostics (AI2D), Perelman School of Medicine University of Pennsylvania Philadelphia Pennsylvania USA; ^7^ Department of Radiology, Perelman School of Medicine University of Pennsylvania Philadelphia Pennsylvania USA; ^8^ Center for Biomedical Image Computation and Analytics (CBICA), Perelman School of Medicine University of Pennsylvania Philadelphia Pennsylvania USA; ^9^ Department of Psychological Sciences University of Connecticut Storrs Connecticut USA; ^10^ Institute for the Brain and Cognitive Sciences University of Connecticut Storrs Connecticut USA; ^11^ Department of Child and Adolescent Psychiatry and Behavioral Science Children’s Hospital of Philadelphia Philadelphia Pennsylvania USA; ^12^ Department of Biostatistics Vanderbilt University Medical Center Nashville Tennessee USA; ^13^ Section on Developmental Neurogenomics National Institute of Mental Health Intramural Research Program Bethesda Maryland USA

**Keywords:** brain networks, brain‐behavior associations, brain‐wide association studies, enrichment, hypothesis testing, intersection–union testing, ordinal dominance curves

## Abstract

Interpreting brain‐behavior relationships through the lens of anatomical parcellations or functional networks is commonplace in human brain mapping. However, statistical approaches for testing whether brain–behavior associations are stronger (i.e., enriched) within a region of interest remain underdeveloped. Here, we propose a permutation‐based approach for network enrichment testing using ordinal dominance curves (NETDOM). In simulation studies, we demonstrate that NETDOM properly controls the type I error rate—unlike other prominent enrichment methods—while exhibiting increased statistical power when enrichment occurs in a subset of in‐network locations. Using data from two large‐scale neurodevelopmental cohorts, we illustrate that NETDOM effectively detects enriched associations between structural and functional brain measures and neurocognitive performance.

## Graphical abstract

NETDOM is a novel statistical framework for testing whether brain–behavior associations within a region of interest are both significantly greater than zero and larger than associations outside of the selected region.

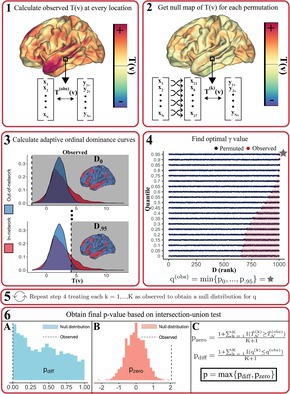

## 1 Introduction

Brain‐wide association studies (BWAS) are a common analytical framework for examining relationships between phenotypes of interest (cognitive functioning, psychopathology, etc.) and brain structure or function (Cheng et al. 2015; Kanai and Rees 2011). However, since mass‐univariate BWAS capture brain‐behavior associations at each location in the image, these studies often suffer from inadequate statistical power after applying a multiple comparison correction (Genon et al. 2022). Numerous suggestions have been made to improve the power of BWAS, including increasing sample sizes (Marek et al. 2022), embracing longitudinal designs (Kang et al. 2024), and using cross‐validated multivariate prediction methods (Spisak et al. 2023). While multivariate models can improve statistical power by leveraging interactions between distributed neural circuits, such methods may lack interpretability and prioritize prediction over unbiased estimation of brain‐phenotype associations. Thus, methods for BWAS that can capture interpretable network‐level effects without sacrificing statistical power are needed to complement multivariate machine learning approaches.

One way to enhance power in univariate BWAS—with parallels in the genomics literature (Reimand et al. 2019)—is through network enrichment testing. Network enrichment tests (NETs) are a class of methods that avoid stringent multiple comparison corrections by aggregating testing statistics for association within predefined brain regions to formally assess whether associations are stronger (i.e., enriched) within a region of interest. An advantage of NETs is that statistical tests are conducted at the level of brain regions or networks that are hypothesized to play an integral role in downstream behavior (Menon and D'Esposito 2022; Smallwood et al. 2021). NETs have been used to formally test for spatial heterogeneity in various contexts, including in brain maps derived from neurotransmitter receptor density (Hansen et al. 2022), intermodal coupling (Baller et al. 2022; Hu et al. 2022), intracortical gray‐matter myelin content (Burt et al. 2020), and functional connectivity (Contreras et al. 2019; Sripada et al. 2014). Moreover, brain parcellations used for NETs have been defined at various spatial scales based on multimodal features of brain tissue such as microarchitecture, topography, structural connectivity, and functional activation (Eickhoff et al. 2018; Glasser et al. 2016).

Preexisting NETs such as the Spin Test (Alexander‐Bloch et al. 2018; Gordon et al. 2016; Vandekar et al. 2015) and Brain Surrogate Maps with Autocorrelated Spatial Heterogeneity (BrainSmash) (Burt et al. 2020) have employed procedures originally designed to test the spatial correspondence between two brain maps. These methods produce null brain–behavior association maps that retain the spatial autocorrelation (SA) present in the original map, enabling comparison of the correlation across locations between the association and binary network partition maps to a null distribution. Other approaches have applied methods from gene set enrichment analysis (Subramanian et al. 2005; Tavazoie et al. 1999; Ziemann et al. 2024) to neuroimaging data sets, either directly (Yao et al. 2020) or by embedding enrichment test statistics within permutation procedures (Eggebrecht et al. 2017; Li et al. 2024; Weinstein et al. 2024; Wheelock et al. 2021). NETs motivated by gene set enrichment analysis typically adapt standard two‐sample hypothesis tests (Fisher's exact test, Kolmogorov–Smirnov test, etc.) to determine whether associations within a region of interest are stronger than those outside that region.

Despite existing methods, at least three key limitations remain unaddressed. First, properly accounting for SA in imaging data remains challenging when conducting NETs. Previous work has demonstrated that both BrainSmash and the Spin Test can suffer from inflated type I error rates when SA is high (Markello and Misic 2021), and in the case of BWAS may not adequately represent the null hypothesis being tested (Weinstein et al. 2024). For instance, if brain–behavior associations do not differ between in‐network and out‐of‐network locations but SA is stronger within versus between networks, both the Spin Test and BrainSmash will likely exhibit inflated false positive rates. Directly applying enrichment methods from genomics may have even graver consequences, since these methods often assume independence between genes (Maleki et al. 2020; Wu and Smyth 2012), which corresponds to independence between locations in neuroimaging contexts. Even procedures that permute phenotype data across participants to generate null brain–behavior maps that account for SA can exhibit downstream issues if the test statistic for comparing in‐network and out‐of‐network associations is not chosen properly. For example, using a test statistic validated in genomic enrichment studies may have suboptimal properties when applied to neuroimaging data sets. In genomics studies, gene–phenotype associations outside the set of interest are plausibly assumed to be centered at zero, while for neuroimaging data in‐network and out‐of‐network associations can be identical but nonzero. When permuting the phenotype, a null distribution of enrichment scores is generated under the setting that in‐network and out‐of‐network associations are equal to zero. Thus, if a test statistic is not identically distributed for all scenarios where in‐network and out‐of‐network associations are equal, inflated false positive rates can occur for situations where in‐network and out‐of‐network associations are identical but nonzero.

Another challenge when conducting enrichment tests is network misalignment. Since NETs use prespecified networks, it is likely that the region where brain‐behavior associations are enriched will not perfectly match the network selected for enrichment testing. Network misalignment typically reduces power as it leads to more null associations falling within the target region. However, misalignment does not impact all test statistics equally, as the upper quantiles of the in‐network association distribution will be less affected by the introduction of null associations in the target region than lower quantiles. It remains unclear whether such challenges could be addressed by data‐adaptive methods that selectively upweight large components of the association vector in the case of neuroimaging settings, as demonstrated previously in studies of rare genetic variants (Pan et al. 2014). If applied successfully, such methods could also mitigate the impact of differences in network granularity across studies (Fornito et al. 2010; Romero‐Garcia et al. 2012).

A third limitation of previous NETs involves the specification of the null hypothesis. While the exact null hypothesis tested by NETs may vary, they can be broadly classified into two categories as “self‐contained” and “competitive” hypothesis tests (Maleki et al. 2020). Self‐contained tests assess whether associations differ significantly from zero within a network of interest, which can be repeated for several networks to obtain a list of network‐specific p‐values. In contrast, competitive tests determine whether there is a difference in association between in‐network and out‐of‐network locations, usually with a simple statistic (e.g., difference in means). A disadvantage of competitive testing frameworks is that null in‐network associations can still lead to statistically significant results if out‐of‐network associations are nonzero, since only differences between in‐network and out‐of‐network associations are captured in the test statistic. Therefore, hybrid methods that identify when in‐network associations are both significantly different than zero and stronger than out‐of‐network associations are desirable. One possible hybrid method is intersection–union testing (Berger and Hsu 1996), which provides a straightforward approach for hypothesis testing when the null hypothesis can be represented as the union of subsets of the parameter space (Benjamini and Heller 2008; Winkler et al. 2016).

In this article, we propose an intersection–union approach for network enrichment testing using ordinal dominance curves (NETDOM). We use ordinal dominance curves (ODCs) to evaluate the stochastic order of in‐network and out‐of‐network associations, as ODCs provide a natural framework for assessing the proportion of out‐of‐network associations that fall below a given percentile of the in‐network association distribution. The main objectives of our method are threefold: First, we strive to account for the spatial correlation across imaging features and control the type I error rate by permuting the phenotype across participants, recalculating a brain‐phenotype association map for each permutation and using a test statistic based on ODCs to compare the stochastic order of in‐network and out‐of‐network associations (Davidov and Herman 2012; Ramdas et al. 2017). Second, to improve statistical power and protect against network misspecification, we employ a data‐adaptive approach and multiply the shifted ODC by a step function that can selectively upweight large values in the association vector. Third, we perform an intersection–union test to ensure that the null hypothesis is rejected only when in‐network associations are both nonzero and stochastically greater than out‐of‐network associations.

## 2 Methods

### 2.1 Null Hypothesis

Similar to Weinstein et al. (2024), let $T\left(v\right)$ denote a measure of association (e.g., Pearson correlation or t‐statistic from a linear regression model) between an imaging metric and outcome of interest at location v, where v=1,…,V indexes locations across the brain. The network or region of interest used for enrichment testing, typically defined from a pre‐specified network map (Yeo et al. 2011), is indicated by $\left\{N\subset 1,\ldots ,V\right\}$. Let $T_{N}$ represent a random variable of association statistics whose realizations are found at a randomly selected location within the network of interest ($T\left(v\right)$ for v∈N), while the realizations of the random variable $T_{N^{c}}$ are located outside the network of interest. For a right‐sided test, NETDOM tests for network enrichment under the null hypothesis

(1) $$H_{0}:\left\{\mathrm{Pr}\left(T_{N}>w\right)\leq \mathrm{Pr}\left(T_{N^{c}}>w\right)\right\}\cup \left\{\mu_{N}\leq \mu^{*}\right\},\forall w\in \left(-\infty ,\infty \right)$$

while for a left‐sided test

(2) $$H_{0}:\left\{\mathrm{Pr}\left(T_{N}<w\right)\leq \mathrm{Pr}\left(T_{N^{c}}<w\right)\right\}\cup \left\{\mu_{N}\geq \mu^{*}\right\},\forall w\in \left(-\infty ,\infty \right)$$

where $\mu_{N}$ represents the expectation of $T_{N}$ and $\mu^{*}$ is the null association value for the test statistic $T\left(v\right)$. In many settings, such as when $T\left(v\right)$ is a t‐statistic from a linear regression model, this corresponds to a setting $\mu^{*}=0$.

Our proposed definition of enrichment differs from previous methods in two main respects. First, we assume that enrichment is directional, such that a one‐sided test is preferred to a more general method that assesses whether $T_{N}$ and $T_{N^{c}}$ are identically distributed. Conducting one‐sided enrichment tests has its advantages, as it improves the interpretability of significant findings and ensures that the null hypothesis is not rejected when in‐network associations are attenuated toward zero. For example, consider a scenario where $\mu_{N}=0.4$ and $\mu_{N^{c}}=0.8$. A two‐sided test may reject the null hypothesis in this setting since in‐network associations are “enriched” in the left direction even though those associations are attenuated toward zero. If one is interested in defining enrichment to encompass both strong positive and negative associations or nonlinear trends, a right‐sided test can be performed using a strictly positive test statistic that only captures the strength of associations and not their direction (e.g., F‐statistic). Additionally, we use an intersection–union test so that the null hypothesis is only rejected when in‐network associations are both significantly greater than zero and out‐of‐network associations (for a right‐sided test).

### 2.2 Network Enrichment Testing Framework

#### 2.2.1 Ordinal Dominance Curves

ODCs provide an intuitive framework for evaluating the stochastic ordering between two random variables. Let X and Y be random variables with cumulative distribution functions F and G, respectively. Then, the ODC is defined as $\mathrm{ODC}\left(t\right)=F\left(G^{-1}\left(t\right)\right)$ where

(3) $$\mathbb{P}\left(X\leq Y\right)=\int_{0}^{1}F\left(G^{-1}\left(t\right)\right)\mathrm{dt}=\int_{0}^{1}\mathrm{ODC}\left(t\right)\mathrm{dt}$$

Thus, the probability that a random draw from Y is greater than a realization from X can be found by integrating over the ODC. Given n and m samples from X and Y respectively, one natural estimator of the ODC uses the plug‐in estimator ${\mathrm{ODC}}_{n,m}\left(t\right)=F_{n}\left(G_{m}^{-1}\left(t\right)\right)$, where $F_{n}$ and $G_{m}$ are empirical cumulative distribution functions. Ramdas et al. (2017) demonstrate that under certain regularity conditions and the null hypothesis that F=G

(4)

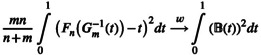

where $\overset{w}{\to }$ denotes weak convergence on the space of probability measures $D\left(\left[0,1\right]\right)$ and B represents a Brownian bridge. Two‐sample hypothesis testing can therefore be performed by comparing the observed test statistic—calculated by properly scaling and integrating over the squared shifted ODC—to a null distribution derived by integrating over a squared Brownian bridge (Tolmatz 2002).

Inspired by Ramdas et al. (2017), we adapt the use of ODCs to compare the distributions of in‐network and out‐of‐network association statistics with three principal contributions. First, since realizations of $T_{N}$ and $T_{N^{c}}$ are not independent due to SA in neuroimaging data, we employ a permutation procedure (described below) and calculate an empirical p‐value instead of using asymptotics. Second, to ensure the null is rejected only when in‐network associations are more extreme, we perform a one‐sided test by integrating over the shifted ODC without squaring. Third, we multiply the shifted ODC by a data‐adaptive step function that allows the inclusion of a subset of statistics within a given network. Note that the power of a given test may depend on the underlying true associations if no uniformly most powerful statistical test exists. For instance, a method that upweights large values when comparing in‐network and out‐of‐network associations may outperform other methods when enrichment is present at only a small subset of in‐network locations. However, that same method may be suboptimal when the entire target region contains enriched associations, due to increased sampling variability at the tails of the distribution. Thus, using an adaptive framework that tailors the test statistic to the underlying data can bolster statistical power across a wide range of potential association patterns (Fan 1996; Pan and Shen 2011; Pan et al. 2014). When using permutation approaches, the test statistic can be tailored to the data in both the observed and permuted samples so that the adaptive procedure does not lead to inflated type I error rates.

#### 2.2.2 NETDOM Overview

**FIGURE 1:**
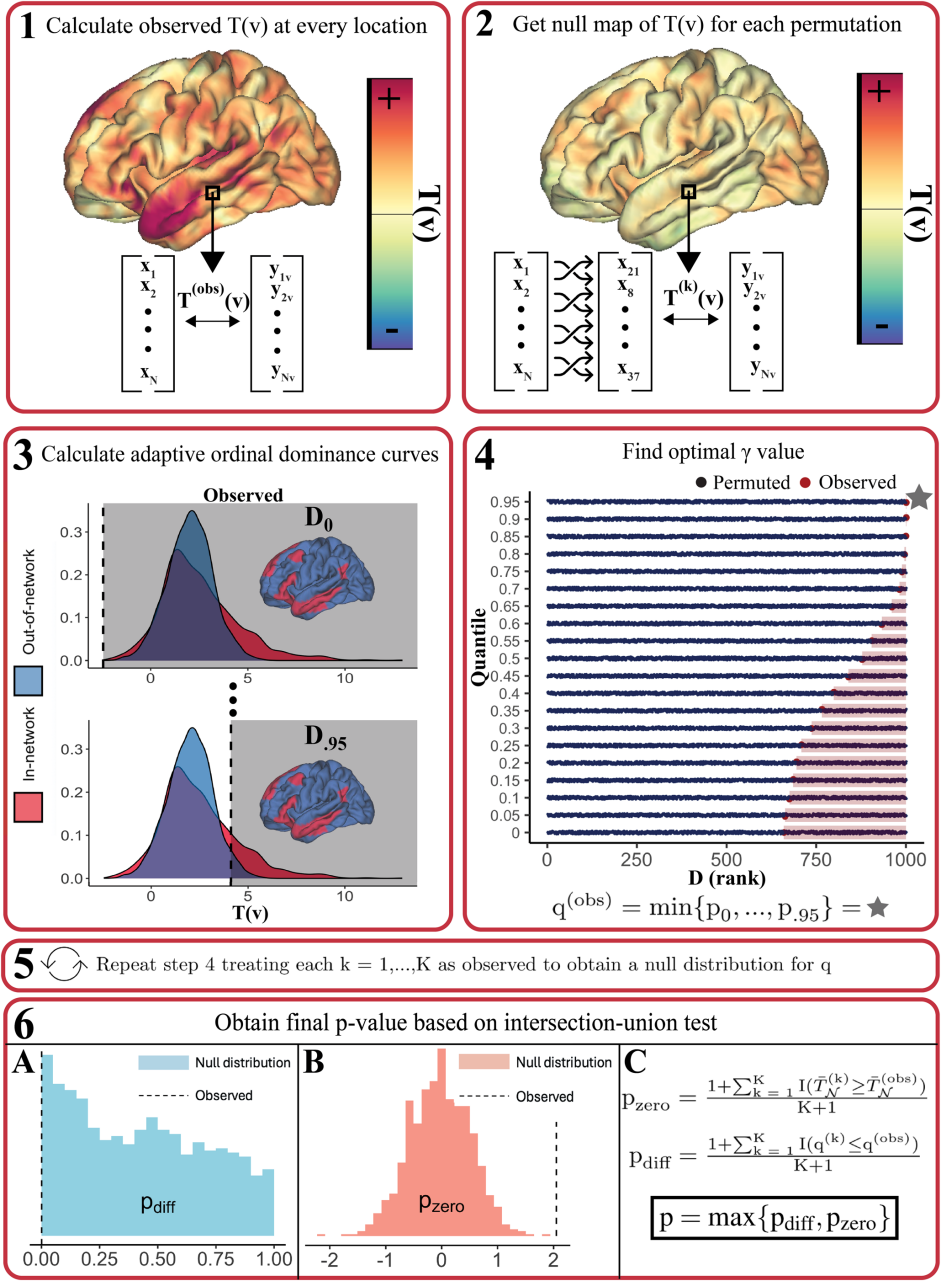
Overview of NETDOM, a network enrichment testing framework that performs an intersection–union test based on shifted ODCs. (1) Test statistics that capture brain‐phenotype associations are computed at v=1,…,V. (2) The phenotype of interest is permuted across participants K times, with (1) repeated on each permuted sample to generate null maps of T(v). (3) A test statistic based on ODCs is calculated to quantify whether in‐network associations are stochastically greater than out‐of‐network associations (see Section 2.2.2 for details). (4) The optimal parameter value for $\gamma \in \left\{0,0.05,..., 0.95\right\}$ is identified based on the data, where γ determines the lowest quantile included when comparing in‐network and out‐of‐network association strengths. (5) A null distribution for q is obtained by finding the optimal γ value for each permuted map so that γ is not overfit. (6) A final p‐value is calculated by taking the maximum of two intermediate p‐values that determine whether in‐network associations are significantly greater than (A) out‐of‐network associations and (B) zero.

Figure 1 contains an overview of our proposed method, NETDOM, which performs network enrichment testing for a prespecified network $N\subseteq \left\{1,\ldots ,V\right\}$ using the following procedure:

1. At each location (v=1,…,V), calculate $T^{\left(\mathrm{obs}\right)}\left(v\right)$, which quantifies the association between the imaging metric and outcome of interest at location v. The test statistic used to calculate $T^{\left(\mathrm{obs}\right)}\left(v\right)$ can be chosen by the researcher, with both signed (t‐statistic, Pearson correlation, etc.) and unsigned (e.g., F‐statistic) association measures allowed.
2. For k=1,…,K randomly permute the phenotype across individuals and repeat step 1, obtaining $T^{\left(k\right)}\left(v\right)$ for each permuted sample. Note that the type of permutation procedure applied will likely depend on the underlying data. For instance, if one is interested in testing for enrichment in the presence of nuisance variables (e.g., a multiple regression model), a Freedman‐Lane procedure can be performed (Freedman and Lane 1983; Winkler et al. 2014) where the same permutation matrix is applied at each location to preserve the SA in the observed image. A stratified permutation approach can also be applied if there is structured dependence between observations (e.g., multiple individuals from the same family) that would violate the exchangeability assumption of standard permutation tests (Winkler et al. 2015).
3. Let $F_{n}^{\left(\mathrm{obs}\right)}$ and $G_{m}^{\left(\mathrm{obs}\right)}$ represent the empirical cumulative distribution functions for $T_{N^{c}}^{\left(\mathrm{obs}\right)}$ and $T_{N}^{\left(\mathrm{obs}\right)}$, where $n=|N^{c}|$, m=∣N∣, and ∣⋅∣ denotes the cardinality of the set. Given a finite set of γ values (e.g., $\gamma =\left\{0,0.05,...,0.95\right\})$, calculate

(5) $$D_{\gamma }^{\left(\mathrm{obs}\right)}=\int_{0}^{1}w_{\gamma }\left(t\right)\left(F_{n}\left(G_{m}^{-1}\left(t\right)\right)-t\right)\mathrm{dt}$$

where

(6) $$w_{\gamma }\left(t\right)=\left\{\begin{array}{cc} 1 & \text{if}\;t>\gamma \\ 0 & \text{otherwise} \end{array}\right.$$

The data‐adaptive parameter γ can be tuned such that only the ${\left(100\times \gamma \right)}^{\mathrm{th}}$ percentile and above are used to test for differences in the in‐network and out‐of‐network associations. Additionally, we denote the sample mean of the in‐network associations as ${\bar{T}}_{N}^{\left(\mathrm{obs}\right)}$. Repeat this procedure on each permuted sample, obtaining $D_{\gamma }^{\left(k\right)}$ and ${\bar{T}}_{N}^{\left(k\right)}$ for k=1,…,K.

4 For a right‐sided test, compute a list of intermediate p‐values using each γ where

(7) $$p_{\gamma }^{\left(\mathrm{obs}\right)}=\frac{1+\sum_{k=1}^{K}I\left(D_{\gamma }^{\left(k\right)}\geq D_{\gamma }^{\left(\mathrm{obs}\right)}\right)}{K+1}$$

and $I\left(\cdot \right)$ is the indicator function. Note that for a left‐sided test, the inequality should be flipped such that lower values of $D_{\gamma }^{\left(\mathrm{obs}\right)}$ correspond with lower p‐values. Next, find the optimal value of γ by calculating

(8) $$q^{\left(\mathrm{obs}\right)}=\min\left\{p_{0}^{\left(\mathrm{obs}\right)},p_{0.05}^{\left(\mathrm{obs}\right)},\ldots ,p_{0.95}^{\left(\mathrm{obs}\right)}\right\}$$

5 Since $q^{\left(\mathrm{obs}\right)}$ is derived by finding the minimum of a list of p‐values, using $q^{\left(\mathrm{obs}\right)}$ directly for hypothesis testing would lead to inflated false positive rates. Therefore, repeat step 4, treating each k=1,…,K as the “observed” map to obtain a null distribution for q, where $p_{\gamma }^{\left(k\right)}=\frac{1}{K}\left[1+\sum_{k_{1}\neq k}I\left(D_{\gamma }^{\left(k_{1}\right)}\geq D_{\gamma }^{\left(k\right)}\right)\right]$ and $q^{\left(k\right)}=\min\left\{p_{0}^{\left(k\right)},p_{0.05}^{\left(k\right)},\ldots ,p_{0.95}^{\left(k\right)}\right\}$. This procedure is similar to previous methods (Pan and Shen 2011; Pan et al. 2014) where permuted samples are leveraged to generate a null distribution for a test statistic adapted to the underlying data.
6 Obtain a final p‐value for an intersection–union hypothesis test by calculating $p=\max\left(p_{\text{diff}},p_{\text{zero}}\right)$ where

(9) $$p_{\text{diff}}=\frac{1+\sum_{k=1}^{K}I\left(q^{\left(k\right)}\leq q^{\left(\mathrm{obs}\right)}\right)}{K+1}$$

and

(10) $$p_{\text{zero}}=\frac{1+\sum_{k=1}^{K}I\left({\bar{T}}_{N}^{\left(k\right)}\geq {\bar{T}}_{N}^{\left(\mathrm{obs}\right)}\right)}{K+1}$$

Again, for a left‐sided test, the inequality is flipped when finding $p_{\text{zero}}$ such that strong negative associations lead to lower p‐values.

#### 2.2.3 Competing Methods

We compare NETDOM to four previously established methods for enrichment testing—Regional Imaging Genetic Enrichment Analysis (Yao et al. 2020), the Spin Test (Alexander‐Bloch et al. 2018), Brain Surrogate Maps with Autocorrelated Spatial Heterogeneity (Burt et al. 2020), and Network Enrichment Significance Testing (Weinstein et al. 2024). We provide a brief description of each method below along with the software used for implementation.

##### 2.2.3.1 Regional Imaging Genetic Enrichment Analysis

Yao et al. (2020) proposed Regional Imaging Genetic Enrichment Analysis (RIGEA) as an extension of previous methods for gene set enrichment analysis to imaging studies. RIGEA performs enrichment testing by thresholding the vector of association statistics and categorizing $T\left(v\right)$ into significant and non‐significant associations. Here, we let $s=\sum_{v\in V}I\left(T\left(v\right)\geq 1.96\right)\cdot I\left(v\in N\right)$ indicate the number of significant in‐network associations and $S=\sum_{v\in V}I\left(T\left(v\right)\geq 1.96\right)$ be the total number of significant associations when conducting right‐sided enrichment tests. Note that a left‐sided test can easily be conducted by defining significant associations as $T\left(v\right)\leq -1.96$ when calculating s and S. The binarized association statistics and network partition can be used to create a 2×2 frequency table, such as the one below:

**Table:** 

	Vertex location		
	In‐network	Out‐of‐network	Total
Significant hits	s	S−s	S
Non‐significant hits	m−s	n−S+s	V−S
Total	m	n	V

Based on a Fisher's exact test on the 2×2 contingency table, a p‐value for enrichment is obtained as

(11) $$p=\sum_{s\leq x\leq S}\frac{\left(\begin{array}{c} S\\ x \end{array}\right)\left(\begin{array}{c} V-S\\ m-x \end{array}\right)}{\left(\begin{array}{c} V\\ m \end{array}\right)}$$

##### 2.2.3.2 Spin Test

To adapt the Spin Test to the setting of enrichment testing, we use the statistic

(12) $$\mathrm{ES}=\frac{1}{m}\sum_{v\in N}T\left(v\right)-\frac{1}{n}\sum_{v\in N^{c}}T\left(v\right)$$

to quantify an enrichment score (ES) that represents the difference in sample means between in‐network and out‐of‐network associations. The Spin Test performs hypothesis tests by employing a rotation procedure, where for each k=1,…,K the spherical projection of the association map is rotated in the x (left–right), y (anterior–posterior), and z (inferior–superior) axis, with the degrees of rotation uniformly sampled between 0 and 360 degrees. To maintain contralateral symmetry, the opposite (i.e., negative) rotation is performed in the y and z direction while the rotation along the x‐axis is the same for both hemispheres. When the medial wall is rotated into the cortex, these values are discarded when calculating statistics for downstream analyses. After calculating the enrichment score on each permuted map, an empirical p‐value for a right‐sided test is computed using:

(13) $$p=\frac{1+\sum_{k=1}^{K}I\left({\mathrm{ES}}^{\left(k\right)}\geq {\mathrm{ES}}^{\left(\mathrm{obs}\right)}\right)}{K+1}$$

Note that a left‐sided test can be performed by reversing the inequality when calculating p. The Python package BrainSpace was used to implement the Spin Test (Vos de Wael et al. 2020).

##### 2.2.3.3 Brain Surrogate Maps With Autocorrelated Spatial Heterogeneity

Similar to the Spin Test, we used BrainSmash to perform enrichment tests by calculating enrichment scores based on the difference in sample means between in‐network and out‐of‐network associations in both observed and null maps. The full procedure BrainSmash uses to produce SA‐preserving null maps is described elsewhere (Burt et al. 2020). In brief, BrainSmash first permutes the values from the original brain map across locations, destroying the autocorrelation in the original map. Next, the permuted map is smoothed and its variogram is regressed onto the variogram from the original map, with the optimal amount of smoothing determined by minimizing the sum of squared errors when regressing the permuted variogram onto the target variogram. The linear transformation from the permuted to target variogram is then used to generate a surrogate map that preserves SA. On each of the null maps generated by BrainSmash, an identical procedure as the Spin Test can be applied to obtain enrichment scores and an empirical p‐value. BrainSmash was implemented using the Python package BrainSmash with random sub‐sampling due to the large number of vertices in the imaging data (see https://brainsmash.readthedocs.io/ for details). Visual inspection of variograms computed on observed and permuted maps revealed that default parameter values for smoothing and kernel functions were sufficient.

##### 2.2.3.4 Network Enrichment Significance Testing

Network Enrichment Significance Testing (NEST) differs from BrainSmash and the Spin Test with regards to the unit of permutation and method of quantifying enrichment. While BrainSmash and the Spin Test generate surrogate brain‐phenotype association maps that preserve SA, NEST permutes individual‐level phenotype data and recalculates brain‐phenotype association maps under the null using the permuted data. To quantify differences between in‐network and out‐of‐network associations, an enrichment score based on a weighted Kolmogorov–Smirnov test statistic (Subramanian et al. 2005; Weinstein et al. 2024) is calculated on both observed and permuted brain‐phenotype association maps, which can be used to obtain an empirical p‐value as before. We implemented this method using the open‐access R package NEST (https://github.com/smweinst/NEST).

### 2.3 Data

To evaluate NETDOM, we used data from two large‐scale neurodevelopmental studies—the Philadelphia Neurodevelopment Cohort (PNC) and Adolescent Brain Cognitive Development (ABCD) Study (Satterthwaite et al. 2016; Volkow et al. 2018).

#### 2.3.1 Adolescent Brain Cognitive Development Study

Participant data were downloaded from the ABCD BIDS Community Collection (Feczko et al. 2021), which contains the baseline cohort of the ABCD study—a large sample of N=11,878 9–10‐year‐olds from 21 sites across the country. Cognitive scores were obtained for seven assessments from the NIH Toolbox (Picture Vocabulary, Flanker Test, List Sort Working Memory Task, Dimensional Change Card Sort Task, Pattern Comparison Processing Speed Task, Picture Sequence Memory Task, and the Oral Reading Test) and two additional tasks (Little Man Task and Rey Auditory Verbal Learning Task). A Bayesian Probabilistic Principal Component Analysis model with varimax rotation was fit to the cognitive data to obtain composite scores on three neurocognitive domains—general cognition, executive functioning, and learning/memory (Thompson et al. 2018). ABCC Collection 3165 neuroimaging data were processed using the ABCD‐BIDS pipeline detailed in Appendix A.1 in Supporting Information. Time series data from resting‐state and three task‐based scans (Monetary Incentive Delay Task, Stop‐Signal Task, and Emotional N‐back Task) were concatenated to maximize scan length for downstream analyses. Personalized functional networks were derived via a nonnegative matrix factorization approach applied to the time series data to generate 17 discrete personalized functional networks in fsLR 32k space (Cui et al. 2020; Li et al. 2017), which were combined into a group‐level network partition by taking the most frequent network label across participants at each vertex (Keller et al. 2023). Cortical thickness values in fsLR 32k space were also downloaded from the ABCD BIDS Community collection, with V=56,839 after removing vertices along the medial wall. Cortical thickness values were harmonized using ComBat to remove site effects while preserving associations between cortical thickness and age, sex, age×sex, general cognition score, executive functioning score, and learning/memory score (Fortin et al. 2017, 2018; Johnson et al. 2007). The final sample included N=8405 participants with observed demographic, neurocognitive, cortical thickness, and fMRI data.

#### 2.3.2 Philadelphia Neurodevelopmental Cohort

The PNC is a community‐based sample of 8–21 year‐olds designed to integrate multimodal neuroimaging, cognitive and clinical phenotypes, and genetics in order to elucidate the relationship between neurodevelopment and psychiatric disorders (Satterthwaite et al. 2016). Participants completed the Penn Computerized Neurocognitive Battery (CNB), a series of 14 cognitive tests which assess performance across a wide variety of neurocognitive domains including executive functioning, memory, verbal and nonverbal reasoning, social cognition, and sensorimotor speed (Gur et al. 2010, 2012). Here, we focus on three summary scores based on standardized accuracy scores—complex cognition, memory, and social cognition—derived from an exploratory 3‐factor analysis model with oblique rotation (Moore et al. 2015). A subset of these participants (N=1445) underwent multimodal neuroimaging, with the advantage that all participants were scanned on the same 3T Siemens TIM Trio whole‐body scanner with no software or hardware changes during the study. The image acquisition protocol used for the PNC has been briefly summarized in Appendix A.2 in Supporting Information, with further details available elsewhere (Satterthwaite et al. 2014). Neuroimaging data were processed with FreeSurfer (version 5.3) and resampled into fsaverage5 atlas space (Fischl et al. 1999). Functional activation during a fractal n‐back memory task, quantified as % change during 0‐back and 2‐back sequences (Ragland et al. 2002), was used as the primary measure of interest. When testing for enrichment in PNC data, a 17‐network atlas based on intrinsic functional connectivity was used, as detailed in Yeo et al. (2011). Vertices along the medial wall were removed, leaving V=18,715 across both hemispheres. Individuals who failed to meet quality control criteria or had a history of health conditions that would impact brain function (e.g., proscribed psychiatric medications or a history of psychiatric hospitalization) were excluded from the sample, leaving N=1018 participants with complete demographic, neurocognitive, and imaging data.

### 2.4 Plasmode Simulations

To compare NETDOM to other frameworks for enrichment testing, we conducted a plasmode simulation study. In plasmode simulations, we fixed the brain‐phenotype association strengths for both in‐network and out‐of‐network regions in order to evaluate type I error rate and statistical power. However, instead of using a parametric distribution (e.g., multivariate Gaussian) to add noise to the simulated imaging values, we randomly sub‐sampled scaled neuroimaging data to ensure realistic SA in simulated images. Since the performance of enrichment tests may depend on the strength of SA (Weinstein et al. 2024), we generated simulated imaging data using sub‐samples of the previously described cortical thickness (low SA) and functional activation (high SA) data.

At every location, values in the imaging data set were standardized by centering and scaling across participants. Thus, each location had mean zero, while the variance differed between the two modalities to resemble the original data ($\sigma^{2}=\left\{0.09,0.36\right\}$ for cortical thickness and functional activation values, respectively). Given a sample of N rows from this standardized matrix, denoted by $\zeta_{N\times V}$, simulated imaging data was generated using the equation

(14) $$Y_{\mathrm{iv}}=\beta_{0}+0.05\cdot x_{1}-0.05\cdot x_{2}+\beta_{3}\cdot x_{3}+\zeta_{\mathrm{iv}}$$

where $\beta_{0}=3$ if ζ is sampled from cortical thickness data ($\beta_{0}=0$ otherwise), $x_{1}∼\text{Bernoulli}\left(0.5\right),x_{2}∼N\left(0,1\right)$, and $x_{3}∼N\left(0,1\right)$ is the predictor of interest. We considered four different settings for $\beta_{3}$ to evaluate the performance of enrichment methods under association structures where (1) in‐network associations are zero but differ from out‐of‐network associations (2) in‐network and out‐of‐network associations are identical (3) all in‐network associations are stronger than out‐of‐network associations and (4) in‐network associations are more variable than out‐of‐network associations:

**Table:** 

Null or alternative?	$\beta_{3}$
Null	= $\left\{\begin{array}{ll} 0 & \text{if}\;v\in N\\ -0.03 & \text{if}\;v\in N^{c} \end{array}\right.$
Null	=0.04
Alternative	= $\left\{\begin{array}{ll} 0.06 & \text{if}\;v\in N\\ 0.04 & \text{if}\;v\in N^{c} \end{array}\right.$
Alternative	∼ $\left\{\begin{array}{cc} \Gamma \left(2,40\right) & \text{if}\;v\in N\\ \Gamma \left(20, 400\right) & \text{if}\;v\in N^{c} \end{array}\right.\;$ $\Gamma \left(a,b\right)$ denotes the Gamma distribution

For each location v, a linear model of the form

(15) $$Y^{\left(v\right)}=\beta_{0}^{\left(v\right)}+\beta_{1}^{\left(v\right)}\cdot x_{1}+\beta_{2}^{\left(v\right)}\cdot x_{2}+\beta_{3}^{\left(v\right)}\cdot x_{3}+\epsilon^{\left(v\right)}$$

was fit to the simulated data where the vector $Y^{\left(v\right)}=\left(Y_{1}^{\left(v\right)},Y_{2}^{\left(v\right)},\ldots ,Y_{N}^{\left(v\right)}\right)$ indicates simulated imaging values across participants at location v. Right‐sided enrichment tests were conducted using the t‐statistic $\hat{T}\left(v\right)=\frac{{\hat{\beta }}_{3}^{\left(v\right)}}{\mathrm{SE}\left({\hat{\beta }}_{3}^{\left(v\right)}\right)}$ with K=1000 permutations. When enrichment methods required the permutation of individual‐level data (NEST and NETDOM), a Freedman‐Lane permutation procedure was used due to the presence of nuisance covariates in the regression model (Freedman and Lane 1983).

**FIGURE 2:**
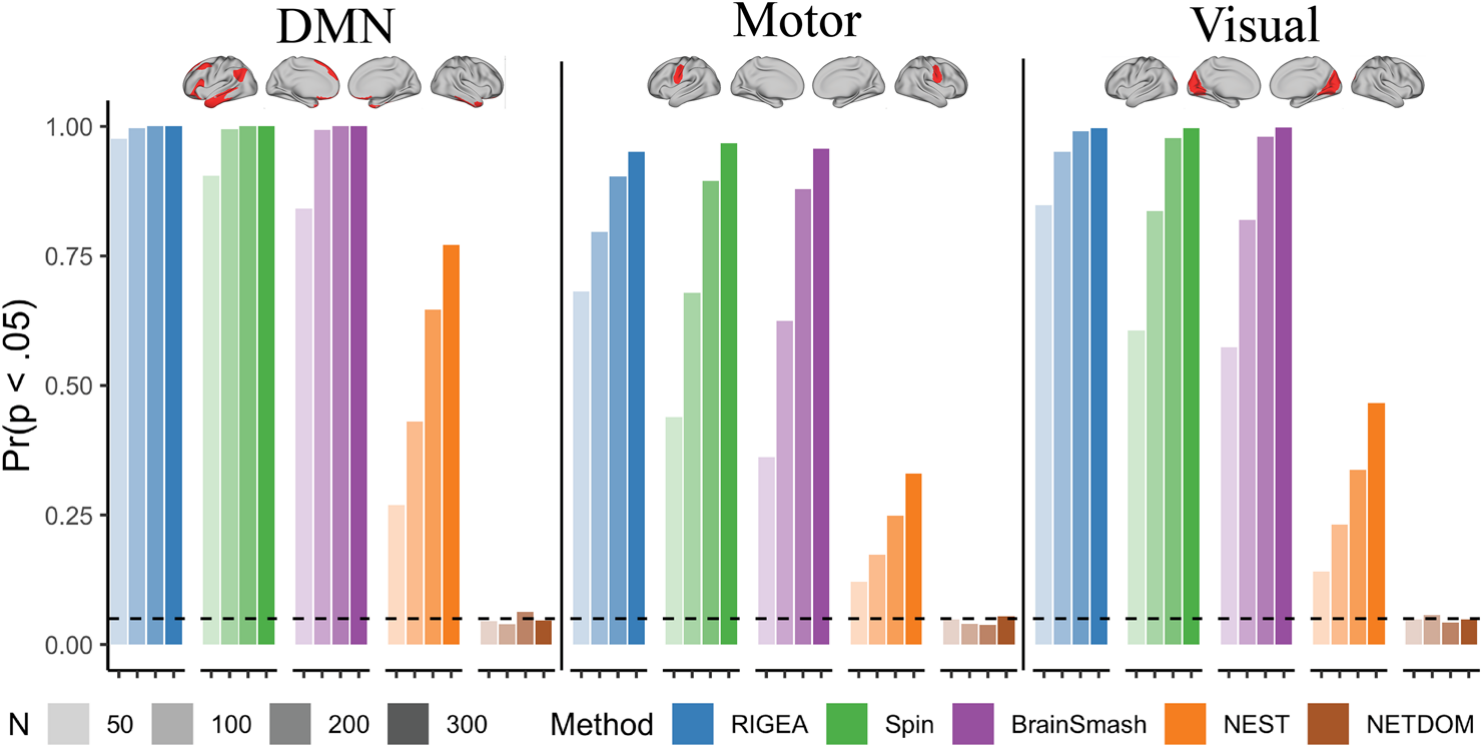
Estimated rejection rates for each proposed method of enrichment testing when $\beta_{3}=0$ for in‐network locations, $\beta_{3}=-0.03$ at out‐of‐network locations, and residuals are sampled from rescaled cortical thickness data (low SA). Results are depicted across sample size (N=50, 100, 200, 300) with K=1000 permutations used to obtain p‐values for right‐sided enrichment tests. The type I error rate is estimated by taking the proportion of 1000 plasmode simulations where p<0.05 for each sample size. Each panel corresponds to a different network used to test for enrichment—default mode (DMN), visual, and motor—which have been depicted on the cortical surface in red (top). Only NETDOM controlled the type I error rate at the nominal level (α=0.05) across all three networks. Increases in sample size were associated with higher rejection rates for all competing enrichment methods.

To examine how the selected target region impacted the performance of each enrichment method, false positive rates and power curves were obtained for three different networks—default mode, motor, and visual—which varied with respect to size, spatial continuity, and location (see Figure 2 for projections of these networks onto the cortical surface). For each unique combination of sample size (N=50, 100, 200, 300), covariance structure (low SA, high SA), network (default mode, motor, visual), enrichment method (RIGEA, Spin Test, BrainSmash, NEST, NETDOM), and $\beta_{3}$ setting, rejection rates were estimated based on the proportion of 1000 simulations where the null hypothesis was rejected at the α=0.05 level.

### 2.5 Data Analysis

After conducting simulation analyses, we applied the previously described enrichment tests to the full ABCD cortical thickness and PNC n‐back data sets to investigate network‐level enrichment in the associations between each respective brain measure and neurocognitive performance. When testing for enrichment in the ABCD data set, linear models of the form

(16) $$\begin{array}{cc} Y^{\left(v\right)}= & \;\beta_{0}^{\left(v\right)}+\beta_{1}^{\left(v\right)}\cdot \mathrm{age}+\beta_{2}^{\left(v\right)}\cdot \mathrm{sex}\\ & \;+\beta_{3}^{\left(v\right)}\cdot \mathrm{age}\cdot \mathrm{sex}+\beta_{4}^{\left(v\right)}\cdot \text{cognitive score}+\epsilon^{\left(v\right)} \end{array}$$

were fit at each location v=1,…,V. The variable “cognitive score” differed between analyses, such that scores on either general cognition, executive functioning, or learning/memory were used for enrichment testing. Previous analyses have reported mixed results on the relationship between cortical thickness and neurocognition in youth samples—likely due to the variability in these associations across age and brain regions (Choi et al. 2024; Schmitt et al. 2019; Shaw et al. 2006). Yet given prior evidence from the ABCD cohort indicating negative associations between cortical thickness and cognitive scores in frontal and parietal regions (Patel et al. 2022), we performed a left‐sided enrichment test for each of the 17 functional networks derived in our sample. Results from exploratory right‐sided enrichment tests are also provided in Appendix B.1 in Supporting Information.

A similar analysis was performed on the n‐back data with some small modifications. First, ${\mathrm{age}}^{2}$ and its interaction with sex were added to the linear models to account for nonlinear effects of age present in the PNC. Second, models fit on the n‐back data focused on different cognitive domains—complex cognition, memory, and social cognition—derived from an exploratory factor analysis model detailed in Section 2.3.2. Third, a right‐sided enrichment test was performed since we hypothesized that higher activation in cortical areas implicated in working memory would correspond with better cognitive performance. For completeness, results from left‐sided enrichment tests are also included in Appendix B.2 in Supporting Information. Fourth, a 17‐network partition (Yeo et al. 2011) was used for enrichment testing, which corresponded closely to the functional networks derived in the ABCD sample. All p‐values were transformed using a false discovery rate (FDR) correction to account for separate hypothesis tests on each of the 17 networks in the partition.

Robustness to network specification was tested by using a previously derived seven‐network parcellation (Yeo et al. 2011) instead of the aforementioned 17 networks in both the ABCD and PNC cohorts. In Appendix B.5 in Supporting Information, we also report NETDOM run times across both sample size (N=50,100,200,300) and feature resolution (V=1000,5000,10000,18715). At first glance, NETDOM may appear to be computationally demanding because association maps are calculated on both observed and permuted data. However, note that the data matrix of non‐imaging covariates does not change across each fitted model, such that an entire association map can be calculated in a small number of matrix operations instead of fitting each model iteratively. Further decreases in run time can be achieved by parallelizing model fitting across permutations.

## 3 Results

### 3.1 Plasmode Simulation Results

As described in Section 2.4, plasmode simulations were generated under four scenarios, which included (1) in‐network associations are zero but differ from out‐of‐network associations (2) in‐network and out‐of‐network associations are identical but nonzero (3) all in‐network associations are larger than out‐of‐network associations and (4) in‐network associations are more variable than out‐of‐network associations. The first two settings correspond to simulations under the null hypothesis that in‐network associations are not greater than zero or out‐of‐network associations, while the last two simulation settings evaluate statistical power when the alternative is true.

#### 3.1.1 Type I Error Rate

Figure 2 displays results for plasmode simulations when $\beta_{3}=0$ for in‐network vertices, $\beta_{3}=-0.03$ at out‐of‐network locations, and residuals are resampled from scaled cortical thickness values (low SA). For each sample size and enrichment method, the type I error rate is estimated as the proportion of 1000 simulations where the null hypothesis is rejected at the α=0.05 level. NETDOM preserved the type I error rate across all sample sizes and networks after accounting for Monte Carlo error, with rejection rates ranging from 3.9% to 6.3%. All competing methods exhibited inflated false positive rates under our proposed null hypothesis for enrichment testing, which were exacerbated when the sample size was large. For example, when N=50 and the default mode network was used as the region of interest, the null hypothesis was rejected in 26.9% of simulations using NEST. However, when N=300, the null hypothesis was rejected in 77.1% of simulations. A similar pattern emerged for visual and motor networks, although false positive rates were generally less extreme. At N=300, NEST rejected the null in 33% of simulations for the motor network and 46.5% for the visual network. An association between sample size and false positive rate emerged for all methods except NETDOM, which showed no such trend. RIGEA, BrainSmash, and the Spin Test exhibited high rejection rates, since these methods only test for contrasts between in‐network and out‐of‐network associations. When N=300, the null hypothesis was rejected in 95.1% or more of simulations for each of these three methods, regardless of the network selected to test for enrichment.

**FIGURE 3:**
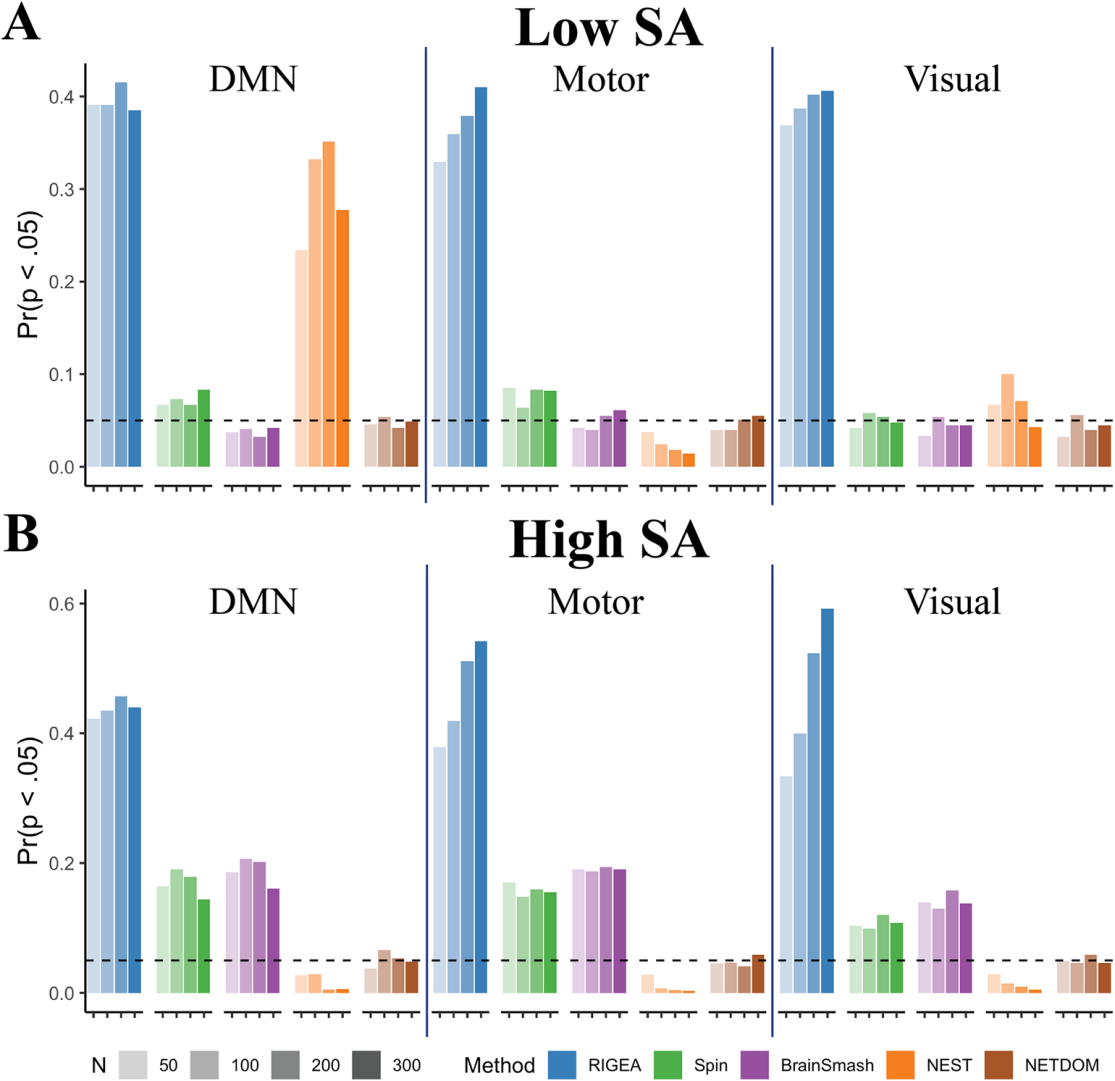
False positive rates from right‐sided enrichment tests when $\beta_{3}=0.04$ at both in‐network and out‐of‐network locations. False positive rates are shown for three separate networks and two different covariance structures based on whether residuals were sampled from rescaled (A) cortical thickness or (B) n‐back activation values. P‐values were obtained using K=1000 permutations for each sample size (N=50,100,200,300). The dashed line represents the rejection cutoff of p=0.05. Only NETDOM controlled the type I error rate across all networks and covariance structures.

Figure 3 contains results for the second null setting, where the true underlying in‐network and out‐of‐network associations are identical but nonzero. RIGEA exhibited inflated false positive rates across all networks and spatial correlation settings, with rejection rates ranging from 32.9% to 59.2%. For the low SA condition, only NETDOM and BrainSmash controlled the type I error rate, with rejection rates only slightly inflated (8.5% at worst) for the Spin Test. However, in high SA conditions, the false positive rate increased substantially for BrainSmash and the Spin Test, such that rejection rates were as high as 20.7% and 19%, respectively. Thus, NETDOM was the only method that controlled the type I error rate across all networks and spatial correlation structures. NEST differed from other methods in that rejection rates were markedly impacted by the network used for enrichment testing. In the low SA setting, NEST yielded false positive rates from 23.4% to 35.1% for the default mode network, whereas using the motor or visual network reduced these rates to 1.4%–3.7% and 4.3%–10%, respectively. Moreover, NEST did not consistently produce inflated false positive rates, as in high SA conditions p‐values were conservative, with rejection rates ranging from 0.3% to 2.9%.

#### 3.1.2 Statistical Power

**FIGURE 4:**
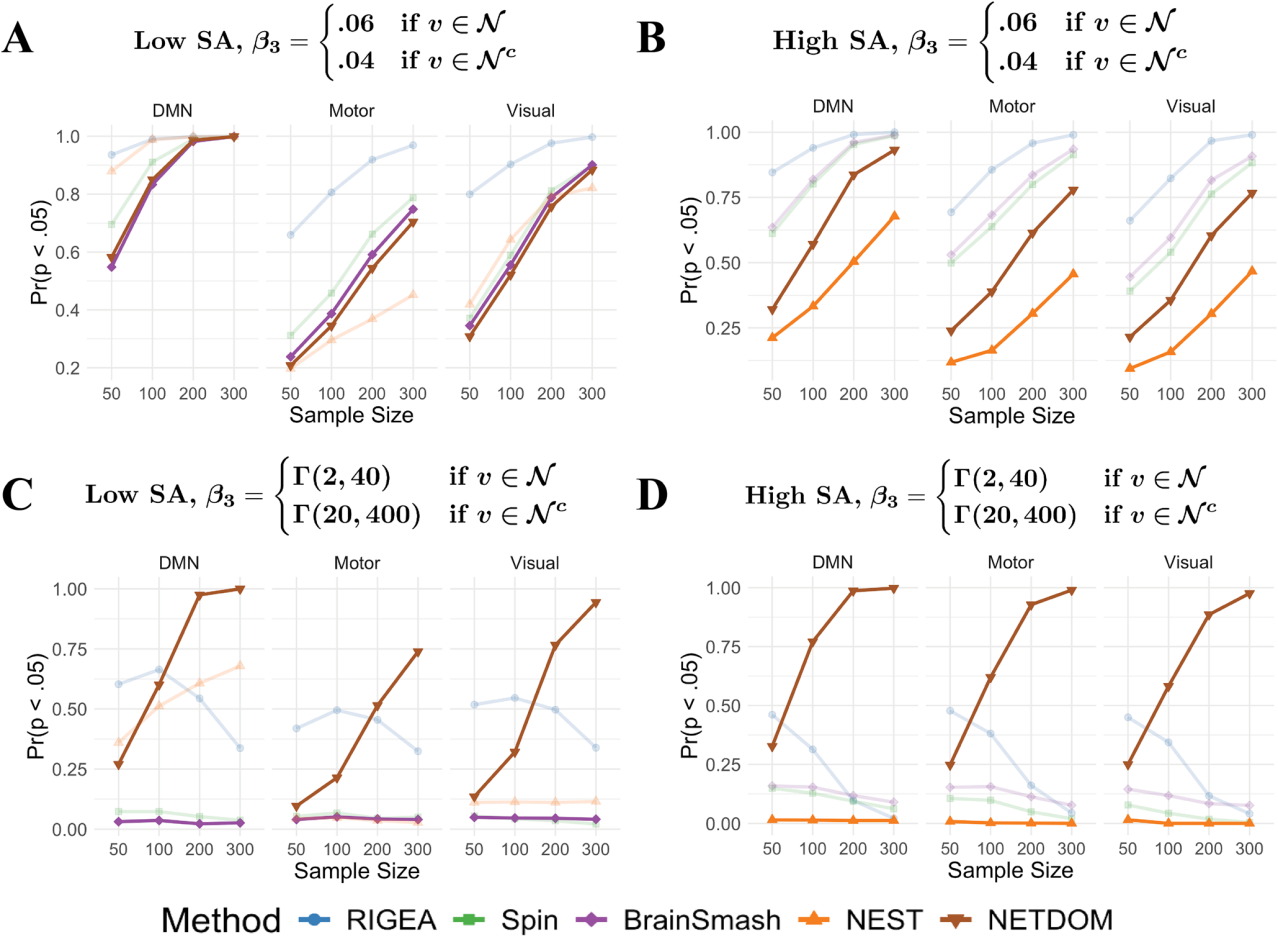
Empirical power was calculated as the proportion of 1000 simulations that the null hypothesis for a right‐sided enrichment test was rejected at the α=0.05 level. Results are reported for three separate networks (see Figure 2), two levels of spatial autocorrelation (see Figure 3), two configurations of in‐network and out‐of‐network association strengths, and four sample sizes (N=50,100,200,300). $\Gamma \left(a,b\right)$ denotes the gamma distribution, such that within each region the true underlying brain‐phenotype associations vary across location. For each simulation, p‐values from enrichment tests were obtained using K=1000 permutations. Lighter shading indicates that the method did not preserve the type I error rate, so statistical power should be interpreted with great caution.

Figure 4 presents simulation results that evaluate the statistical power of NETDOM and other competing methods under the last two simulation scenarios. Since power comparisons are only meaningful when type I error is appropriately controlled, methods with inflated false positive rates are indicated with lighter shading. NETDOM's power increased consistently with sample size across all simulation settings. In the low SA condition, when in‐network associations were stronger than all out‐of‐network associations, NETDOM and BrainSmash exhibited comparable power across sample sizes and networks. However, when in‐network associations had an identical mean but greater variability than out‐of‐network associations, NETDOM consistently outperformed BrainSmash, with greater improvements at large sample sizes. For example, in the default mode network at N=50, empirical power was 27% for NETDOM and 3% for BrainSmash; at N=300, rejection rates were 100% and 2.6%, respectively. This pattern held across networks, though statistical power was generally higher when using the default mode network for enrichment tests, likely due to its larger size.

In the high SA setting, NETDOM had greater empirical power than NEST regardless of sample size, network used for enrichment testing, or spatial variability in association strengths. For example, when N=300 and all in‐network associations were larger, empirical power estimates for NEST ranged between 45.6%–67.8%. However, when using NETDOM, statistical power rose to 76.7%–93.2%. Similar to the low SA condition, improvements in power were largest when in‐network and out‐of‐network associations had identical means but differing variances. When N=200, NETDOM had empirical power higher than 88.6% for all three networks while NEST never rejected the null more than 1.2% of the time.

### 3.2 Data Analysis Results

**FIGURE 5:**
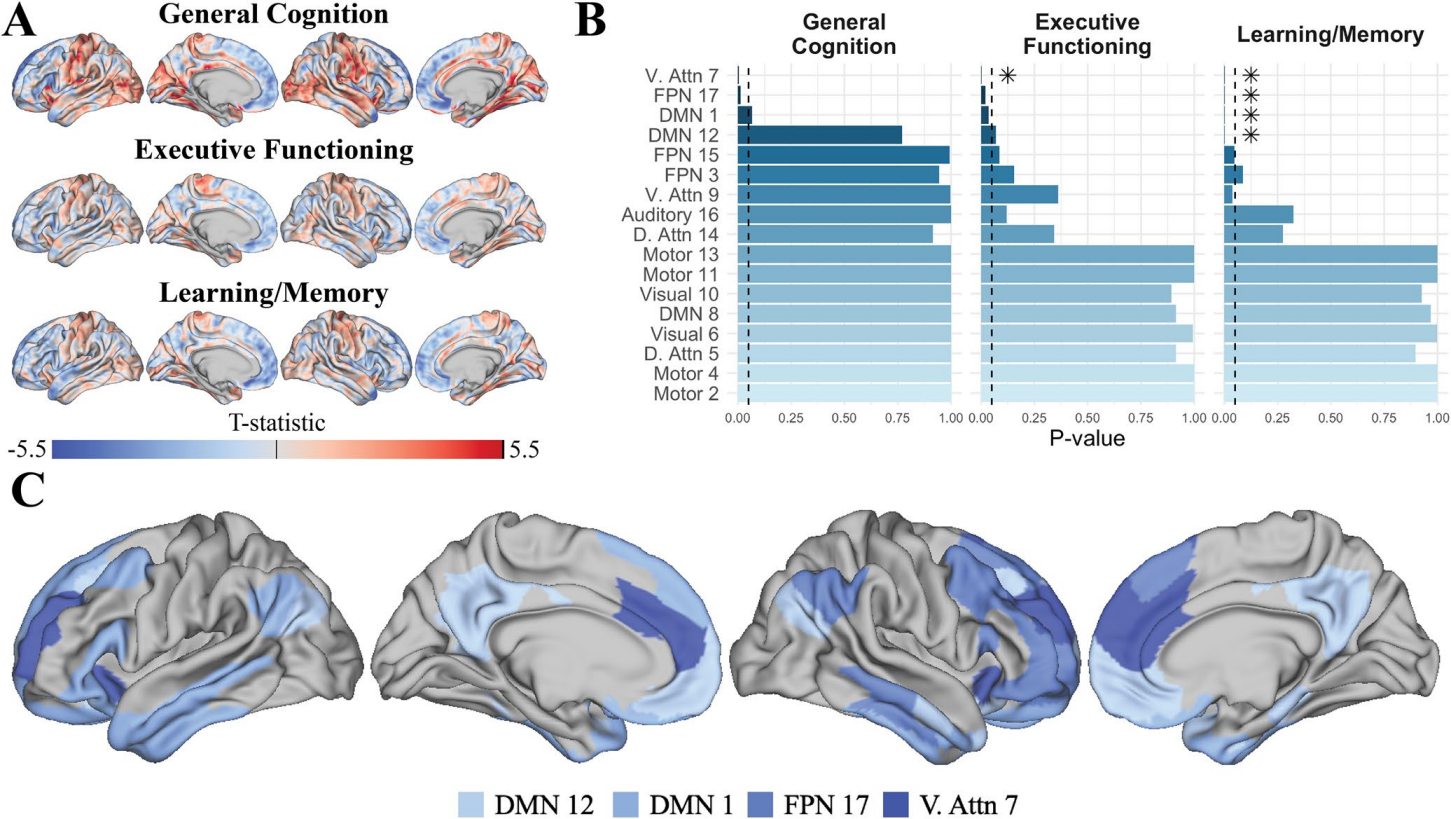
(A) Observed map of t‐statistics from regression models that capture the association between cortical thickness and three neurocognitive scores related to general cognition, executive functioning, and learning/memory. Regression models also controlled for age, sex, and age×sex. (B) Uncorrected p‐values obtained from NETDOM when assessing whether negative associations were enriched in 17 functional networks. The dashed line represents a p=0.05 cutoff, with stars indicating networks that remained significant after applying a FDR correction. (C) Functional networks where enriched associations were detected, represented on the cortical surface (DMN = default mode network; FPN = frontoparietal network; V. Attn = ventral attention).

Figure 5 displays results from applying NETDOM to the Adolescent Brain Cognitive Development Study to assess whether negative associations between cortical thickness and cognitive scores were enriched across 17 functional networks and three neurocognitive domains (see Section 2.3.1). Enrichment tests for associations between learning/memory scores and cortical thickness were significant after applying a FDR correction for the ventral attention, frontoparietal, and default mode network ($p_{\mathrm{adj}}=0.004$ for all networks). Additionally, the ventral attention network remained significant when using executive functioning instead of learning/memory scores ($p_{\mathrm{adj}}=0.034$). While none of the enrichment tests for general cognition survived FDR correction, trends were similar to other neurocognitive domains, with ventral attention (p=0.004) and frontoparietal (p=0.012) networks exhibiting stronger negative associations with cortical thickness. Across the three neurocognitive scores, 84.3% of NETDOM enrichment tests set the tuning parameter to γ=0, suggesting that in most settings optimal power was achieved by integrating over all quantiles when comparing in‐network and out‐of‐network associations (Appendix B.8 in Supporting Information). When using a seven network partition (Yeo et al. 2011), enrichment tests were only significant for learning/memory scores, with enrichment detected in frontoparietal, default, and limbic regions (Appendix B.3 in Supporting Information). Thus, while the 17 network parcellation yielded greater specificity (e.g., no enrichment in superior temporal or rostral middle frontal regions), the spatial correspondence of enriched regions remained high when comparing NETDOM results derived from the seven and 17 network partitions.

Exploratory right‐sided tests illustrated that across neurocognitive scores, positive associations between cortical thickness and neurocognition were enriched in a wide array of motor and visual regions (Appendix B.1 in Supporting Information). Positive enrichment in dorsal attention ($p_{\mathrm{adj}}=0.014$) and default mode ($p_{\mathrm{adj}}=0.012$) networks was also present but only when general cognition scores were the outcome of interest. Results from NETDOM generally corresponded with other enrichment testing frameworks, with some notable exceptions (Appendix B.6 in Supporting Information). NETDOM detected additional enrichment in dorsal attention and visual networks relative to BrainSmash, while no tests remained significant after an FDR correction for the Spin Test. However, applying NEST yielded the same set of enriched networks as NETDOM.

**FIGURE 6:**
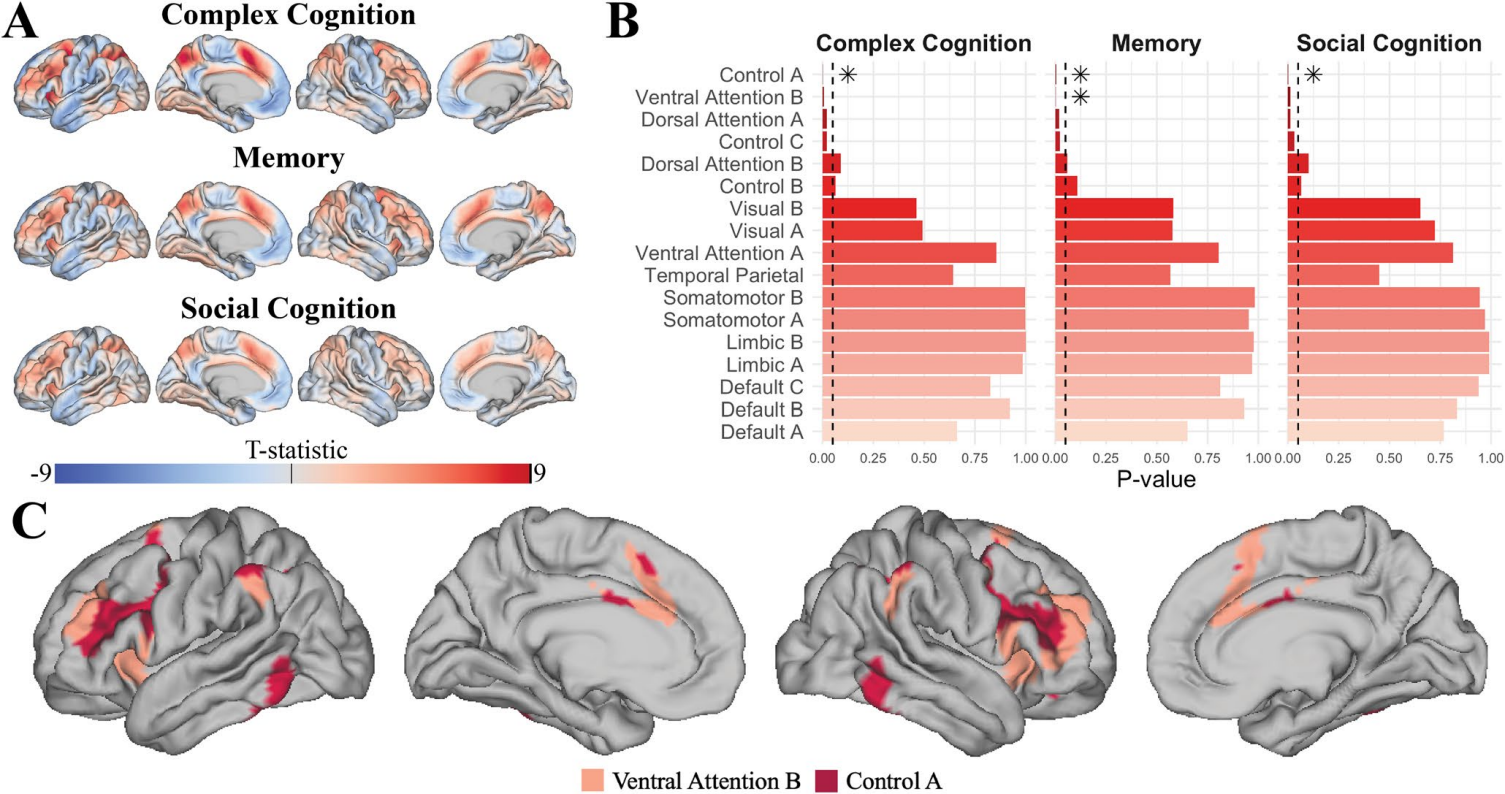
(A) Observed map of t‐statistics from regression models that capture the association between functional activation during a n‐back working memory task and scores on three neurocognitive domains. Regression models also included age, ${\mathrm{age}}^{2}$, sex, age×sex, and ${\mathrm{age}}^{2}\times \mathrm{sex}$ as nuisance covariates. (B) Uncorrected p‐values obtained from NETDOM when assessing whether associations in (A) were enriched in 17 functional networks. The dashed line represents the p=0.05 cutoff, with stars indicating networks that remained significant after a false discovery rate correction was applied across the 17 networks. (C) Functional networks where enriched associations were detected, depicted on the cortical surface.

We also ran NETDOM on the Philadelphia Neurodevelopmental Cohort, testing for network enrichment in associations between functional activation during an n‐back working memory task and neurocognitive scores for 17 functional networks and three cognitive domains (Moore et al. 2015). Across the three neurocognitive scores, NETDOM selected an optimal tuning parameter of γ=0 approximately 75% of the time, suggesting that power is often maximized by incorporating all quantiles when comparing in‐network and out‐of‐network associations (Appendix B.9 in Supporting Information). As illustrated in Figure 6, after applying a false discovery rate correction across networks, enrichment was detected in a frontoparietal control network for complex cognition ($p_{\mathrm{adj}}=0.017$), memory ($p_{\mathrm{adj}}=0.042$), and social cognition scores ($p_{\mathrm{adj}}=0.017$). For a ventral attention network encompassing lateral prefrontal, cingulate, and inferior parietal cortex, enrichment tests were statistically significant only when memory scores were the outcome of interest ($p_{\mathrm{adj}}=0.017$). However, results were consistent across neurocognitive domains, with adjusted p‐values near the significance threshold for complex ($p_{\mathrm{adj}}=0.051$) and social ($p_{\mathrm{adj}}=0.062$) cognition. Overall, uncorrected p‐values suggest that a wider set of ventral attention, dorsal attention, and frontoparietal control networks may be worth exploring in future work.

Results were fairly robust to network specification, as enrichment was detected in dorsal attention and frontoparietal networks for all three neurocognitive scores when using a seven network partition (Appendix B.4 in Supporting Information). While using a seven network parcellation highlighted dorsal instead of ventral attention networks, enriched networks were still primarily located in lateral prefrontal, parietal, and inferior temporal cortex. Left‐sided enrichment tests found no differentially strong negative associations between activation during a working memory task and neurocognitive scores (Appendix B.2 in Supporting Information). Compared to other enrichment tests, NETDOM exhibited greater network specificity—failing to reject the null for a wide range of ventral and dorsal attention, somatomotor, and limbic regions that were statistically significant when using other approaches (Appendix B.7 in Supporting Information). However, NETDOM did perform comparably to NEST, with the exception that only NEST detected enrichment in the dorsal attention network. Appendix B.5 in Supporting Information presents the runtime of NETDOM on a single computing core while varying sample size and vertex resolution, with median runtimes around 7.5 min for neuroimages in fsaverage5 space with N=300 participants.

## 4 Discussion

NETDOM is a novel method for assessing whether brain–phenotype associations are enriched within a predefined region of interest. The development of NETDOM was guided by three primary objectives. First, NETDOM integrates both self‐contained and competitive testing frameworks to identify networks where in‐network associations exceed both zero and out‐of‐network associations. Second, our method accommodates varying levels of spatial autocorrelation common in multimodal neuroimaging data via a permutation procedure, such that the type I error rate is controlled. Third, NETDOM uses an adaptive test statistic that preserves power even when enrichment occurs in only a part of the target region.

Plasmode simulations illustrated that NETDOM controlled the type I error rate when in‐network associations were null, unlike all other competing enrichment methods (Figure 2). Previous test statistics used in enrichment tests, such as the weighted Kolmogorov–Smirnov test statistic employed by NEST (Subramanian et al. 2005; Weinstein et al. 2024), have attempted to avoid rejecting the null in this situation by down‐weighing associations closer to zero. However, when in‐network and out‐of‐network associations differ significantly, using a weighted Kolmogorov–Smirnov test statistic will still lead to inflated false positive rates (Maleki et al. 2020). Thus, intersection–union tests provide an appealing framework for properly controlling the type I error rate in these settings. More broadly, our framework improves the rigor of enrichment tests, which have often relied on post hoc comparisons of self‐contained statistical tests to determine whether associations are enriched in a given region (Hackman et al. 2021; Patel et al. 2022; Shaw et al. 2006).

Plasmode simulations further demonstrated the importance of properly taking into account the SA present in neuroimaging data. RIGEA, a method that directly applies Fisher's exact test to thresholded brain‐phenotype associations, exhibited inflated false positive rates across all null settings where association strengths did not differ across networks. Our results corroborated previous findings that enrichment methods which resample brain‐phenotype association maps—such as BrainSmash and the Spin Test—fail to control the type I error rate when SA is high (Markello and Misic 2021; Weinstein et al. 2024). These results suggest that such methods are suboptimal when SA is stronger within networks than between them, which is common in neuroimaging data since regions of interest are often spatially continuous. Furthermore, we observed that NEST, a method designed to address this limitation, performs poorly when in‐network and out‐of‐network associations are identical but nonzero. Whether NEST was conservative or anti‐conservative in this scenario depended on the interaction between SA and the region selected for enrichment testing. Poor performance resulted from the weighting scheme used by NEST, which can cause large association values to be weighted differently in permuted and observed data—even under the null—which impacts downstream enrichment scores. Thus, NEST should be used only when out‐of‐network associations can be plausibly assumed to be zero. These findings underscore the importance of developing test statistics whose null distribution remains constant for all settings where in‐network and out‐of‐network associations are identical.

Further simulation analyses suggested that in cases where the true underlying in‐network associations were more variable than out‐of‐network associations, NETDOM maintained much higher power than competing methods (Figure 4). Additionally, in settings where all in‐network associations were enriched, NETDOM exhibited similar power to other methods that properly controlled the false positive rate. Thus, adaptive methods originally proposed in the genomics literature (Pan and Shen 2011; Pan et al. 2014; Price et al. 2010) can be applied in neuroimaging contexts to preserve statistical power for a wide variety of situations where the alternative hypothesis is true. These approaches may enhance the replicability of enrichment analyses by reducing variability attributable to differences in network scale across studies (Fornito et al. 2010; Romero‐Garcia et al. 2012).

When applying NETDOM to two large neurodevelopmental cohorts, results largely mirrored previous findings. In the Philadelphia Neurodevelopment Cohort, associations between functional activation during a n‐back task and neurocognitive scores were enriched in both control and ventral attention networks (Figure 5). These networks have a notable presence in lateral and superior frontal, supramarginal, and cingulate regions, which have been previously implicated in working memory tasks (Deschamps et al. 2014; Funahashi 2017; Lara and Wallis 2015; Lenartowicz and McIntosh 2005; Nissim et al. 2017; Salmon et al. 1996). For the Adolescent Brain Cognitive Development Study, negative associations between cortical thickness and neurocognition were enriched in ventral attention, frontoparietal, and default mode networks (Figure 6). These findings build upon previous studies by performing a formal statistical test when investigating whether inverse correlations between cortical thickness and cognition are stronger in frontal and parietal association cortices for young adolescents (Goh et al. 2011; Patel et al. 2022; Schmitt et al. 2019).

For a majority of enrichment tests performed in both the ABCD and PNC, NETDOM selected a tuning parameter of γ=0, suggesting that in most circumstances optimal power is achieved by integrating over all quantiles when comparing in‐network and out‐of‐network associations. While these results coincide with the intuition that integrating over all quantiles leads to a more stable estimator of differences in distribution between in‐network and out‐of‐network regions, data set properties could also play a key role. For example, in both the ABCD and PNC, enrichment was detected in multiple regions, such that the upper quantiles of the out‐of‐network associations are disproportionately composed of vertices from other enriched networks. Thus, it may be worthwhile to explore enrichment tests where in‐network associations are compared to a prespecified null region as opposed to all other locations in the brain.

While the present study focused on two imaging metrics—cortical thickness and activation during an n‐back working memory task—future work could easily extend NETDOM to additional modalities. For instance, common white‐matter tractography atlases (Hua et al. 2008; Wakana et al. 2007) could be used to perform enrichment tests for metrics derived from diffusion tensor imaging. NETDOM could even be applied to functional connectivity analyses by grouping functional connectivity values based on the network label of both vertices as in Sripada et al. (2014).

The present study has some notable limitations that could also be addressed in future work. First, while NETDOM can accommodate any association measure when performing enrichment tests, we evaluated our method using t‐statistics from linear regression models. It may be worth exploring how NETDOM performs when nonlinear approaches such as generalized additive models are used to examine enrichment in brain‐phenotype associations.

Second, NETDOM was evaluated using association statistics from mass‐univariate models. Yet, researchers have increasingly advocated for multivariate approaches when conducting BWAS (Habeck and Stern 2010; Haxby 2012; Peelen and Downing 2023; Spisak et al. 2023). It should be noted that NETDOM does not have any intrinsic requirement that associations are derived from mass‐univariate models. Li et al. (2024) offer a promising framework for extending permutation‐based enrichment tests from univariate to multivariate settings that could be applicable to NETDOM as well.

Third, in our simulation study, we considered only one setting where association strengths varied across locations within a network of interest. However, the spatial distribution of the true underlying brain–phenotype associations within a network may vary depending on the imaging modality and outcome of interest. Future studies could therefore investigate more complex spatial distributions of association strengths. It remains to be seen whether hierarchical frameworks that model within‐network variability can be adapted for enrichment tests with strong spatial autocorrelation (Ma et al. 2020).

Fourth, we considered a straightforward approach—using a step function—for adapting our test statistic to the underlying data. While this method has the benefit of interpretability, weight functions that more flexibly upweight quantiles where in‐network and out‐of‐network associations differ could further increase statistical power.

Fifth, our approach relies on an empirical estimator of the cumulative distribution function to compare the distribution of in‐network and out‐of‐network associations. However, if the number of in‐network locations is small (< 100), such estimators could become noisy and may underperform relative to measures of central tendency. Thus, future work could explore how NETDOM performs in settings where the network parcellation becomes increasingly granular and the spatial resolution is low.

Last, we considered an intersection–union test to ensure that the null hypothesis was rejected only when in‐network associations significantly differed from both zero and out‐of‐network associations. While intersection–union tests provide an intuitive framework for formerly defining enrichment, such tests can be particularly stringent by taking the maximum of the *p*‐values derived from each individual test. Thus, future work could explore other methods for joint inference (Winkler et al. 2016) that combine *p*‐values from multiple hypothesis tests without requiring both to be significant in order for the null hypothesis to be rejected.

Overall, NETDOM overcomes key limitations of previous enrichment tests for brain–phenotype associations by appropriately controlling the type I error rate while offering greater statistical power when enrichment is present at a subset of in‐network locations.

## Author Contributions

**Noah Hillman:** conceptualization, methodology, software, data curation, writing (original draft), reviewing, and editing. **Sarah M. Weinstein:** conceptualization, methodology, software, data curation, writing, reviewing, and editing. **Joëlle Bagautdinova:** data curation, writing, reviewing, and editing. **Kevin Y. Sun:** data curation, writing, reviewing, and editing. **Matthew Cieslak:** data curation, writing, reviewing, and editing. **Taylor Salo:** data curation, writing, reviewing, and editing. **Yong Fan:** writing, reviewing, and editing. **Arielle S. Keller:** data curation, writing, reviewing, and editing. **Aaron F. Alexander‐Bloch:** methodology, software, writing, reviewing, and editing. **Simon N. Vandekar:** methodology, writing, reviewing, and editing. **Armin Raznahan:** writing, reviewing, and editing. **Theodore D. Satterthwaite:** conceptualization, methodology, data curation, writing, reviewing, and editing. **Haochang Shou:** supervision, conceptualization, methodology, writing, reviewing, and editing. **Russell T. Shinohara:** supervision, conceptualization, methodology, writing, reviewing, and editing.

## Funding

This work was supported by the National Institute of Mental Health (R01MH123550, R01MH112847, R01MH113550, R37MH125829, 1ZIAMH002949, R01MH123563, 1L30MH131061‐01, R01MH132934, R01MH134896, R01MH133843), the National Institute of Neurological Disorders and Stroke (R01NS112274, U24NS130411), the National Institute of Biomedical Imaging and Bioengineering (R01EB022573), the Brain and Behavior Research Foundation, NARSAD Young Investigator Award National Institute on Aging, R01AG066650.

## Conflicts of Interest

Aaron F. Alexander‐Bloch holds equity in Centile Biosciences Inc. Russell T. Shinohara has received consulting income from Octave Bioscience and compensation for scientific reviewing from the American Medical Association.

## Supporting information


**Data S1:** Supporting Information.

## Data Availability

Neuroimaging and behavioral data were obtained from the Philadelphia Neurodevelopmental Cohort (PNC) and Adolescent Brain Cognitive Development (ABCD) study. Access to PNC data can be requested at https://www.ncbi.nlm.nih.gov/projects/gap/cgi‐bin/study.cgi?study_id=phs000607.v3.p2. ABCD data used in this study was acquired from the NIMH Data Archive (NDA) at https://abcdstudy.org. ABCD Study data is available to investigators with an approved NDA Data Use Certification (DUC). Code is available at https://github.com/Nhillman19/NETDOM.
