# Supplementary material for: Testing for Network Specificity in Brain‐Behavior Associations Using Ordinal Dominance Curves
Source: Hum Brain Mapp. 2026 Mar 23;47(5):e70493. doi: 10.1002/hbm.70493 (PMC13081700; doi:10.1002/hbm.70493)
Supplement: Supplementary file 1 — Data S1: Supporting Information. [file HBM-47-e70493-s001.pdf]

Appendix

A. Neuroimaging Data Preprocessing

A.1 Adolescent Brain Cognitive Development Study

Details on data preprocessing for the ABCD-3165 collection are available elsewhere (https://collection3165.readthedocs.io/en/stable/). Briefly, DICOMs in the ABCD-3165 collection that passed initial quality control measures were converted to BIDS using the `abcd-dcm2bids` wrapper (https://github.com/ABCD-STUDY/abcd-dicom2bids). Neuroimaging data was processed according to the ABCD-BIDS processing pipeline, an updated version of the Human Connectome Project (Glasser et al., 2013) MRI procedure. The ABCD-BIDS pipeline can be broken down into three structural stages (PreFreesurfer, Freesurfer and PostFreesurfer) and three functional stages (volume, surface, and preprocessing). In the PreFreeSurfer stage, T1w images were skull stripped, denoised, and bias corrected using the Advanced Normalization Tools (https://stnava.github.io/ANTs/). Structural T1w images were then processed through FreeSurfer, which performs tissue segmentation and reconstructs white and pial cortical surfaces in standard FreeSurfer atlas space. These surfaces were registered to the Conte69 template using ANTs SyN non-linear registration and CIFTI grayordinates were generated. For fMRI data, functional runs (resting-state and task fMRI) were processed using the DCAN/HCP pipeline. First, distortions from gradient nonlinearities were removed from EPIs and each volume was realigned to the first frame using FSL's FLIRT (six degrees of freedom) to correct for head motion. The single-band reference image was then registered to the T1w, with this registration being used to align all fMRI volumes to anatomical data. All individual fMRI volumes were additionally registered to MNI space and masked. The volume time series were projected to the standard grayordinate CIFTI space prior to DCAN BOLD Processing (DBP). DBP performed nuisance regression and filtering: fMRI timeseries were demeaned and detrended, then regressed against confounds for global signal trend and movement. The residual BOLD time series were bandpass filtered (0.008–0.09Hz) using a 2nd-order Butterworth filter, a DBP respiratory motion filter (18.582–25.726 breaths per minute) was applied, and frames exceeding a framewise displacement threshold were identified for censoring (.2mm or +/- 3 standard deviations). Finally, parcellated timeseries were generated for common atlases (Glasser et al., 2016; Gordon et al., 2016; Power et al., 2011; Yeo et al., 2011).

A.2 Philadelphia Neurodevelopment Cohort

Details on neuroimaging acquisition parameters for the Philadelphia Neurodevelopment Cohort have been published previously (Satterthwaite et al., 2014). All scans were acquired on a single Siemens Tim Trio 3T scanner with a 32-channel head coil at the Hospital

of the University of Pennsylvania. A single T1w image was obtained using an magnetization prepared, rapid-acquisition gradient-echo (MPRAGE) sequence (TR = 1810ms, TE = 3.5ms, 0.9×0.9×1.0 mm voxel resolution) with Siemens `prescan normalize` enabled to minimize receive coil shading. All T1 images were visually inspected to ensure high quality. T1w images were processed with FreeSurfer (version 5.3), which included normalization, registration, and projection of cortical surfaces onto the fsaverage5 template. BOLD images were acquired with a single-shot, interleaved multi-slice, gradient-echo, echo planar imaging (GE-EPI) sequence (TR = 3000ms, TE = 32ms, 3×3×3 mm voxel resolution). fMRI time series data was processed using the eXtensible Connectivity Pipeline (XCP) Engine to minimize artifacts due to excessive head motion (Ciric et al., 2018). Participants completed a fractal n-back task, which involved showing geometric figures (fractals) for 500ms, after which an interstimulus interval of 2500ms occured. The task involved three conditions – 0-back, 1-back, and 2-back. For the 0-back condition, participants were instructed to press a button if a prespecified fractal appeared; in the 2-back condition, participants pressed the button if the presented fractal was identical to the one shown two trials ago. In this analysis, we quantified working memory load as the voxel-level percent change in brain activation between the 0-back and 2-back conditions (Ragland et al., 2002).

B. Supplemental Results

B.1 Right-sided Enrichment Test – ABCD

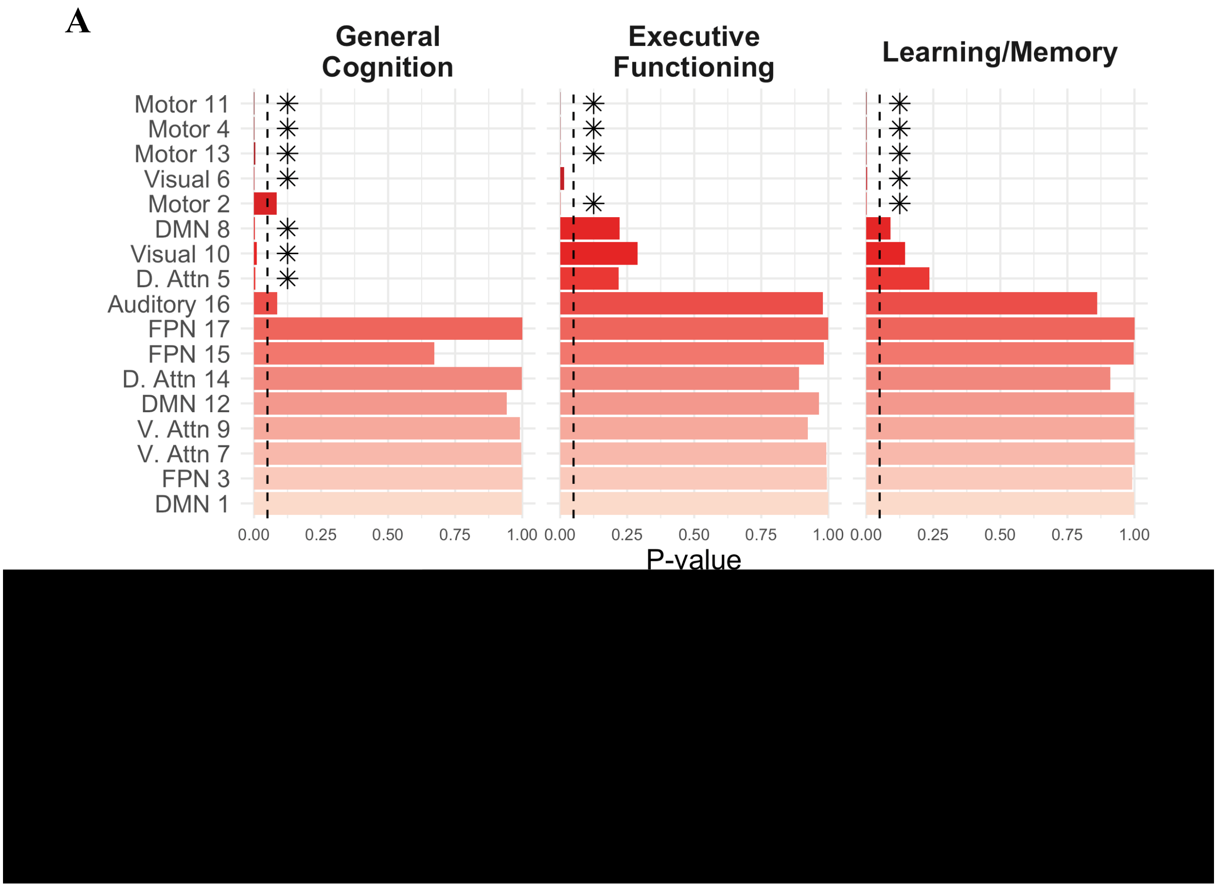

Figure 7: (A) Uncorrected p-values obtained from NETDOM when assessing whether positive associations between cortical thickness and neurocognitive scores were enriched in seventeen functional networks. The dashed line represents a $p = .05$ cutoff, with stars indicating networks that remained significant after applying a FDR correction. (B) Functional networks where enriched associations were detected (DMN = Default Mode Network; D. Attn = Dorsal Attention).

B.2 Left-sided Enrichment Test – PNC

1166

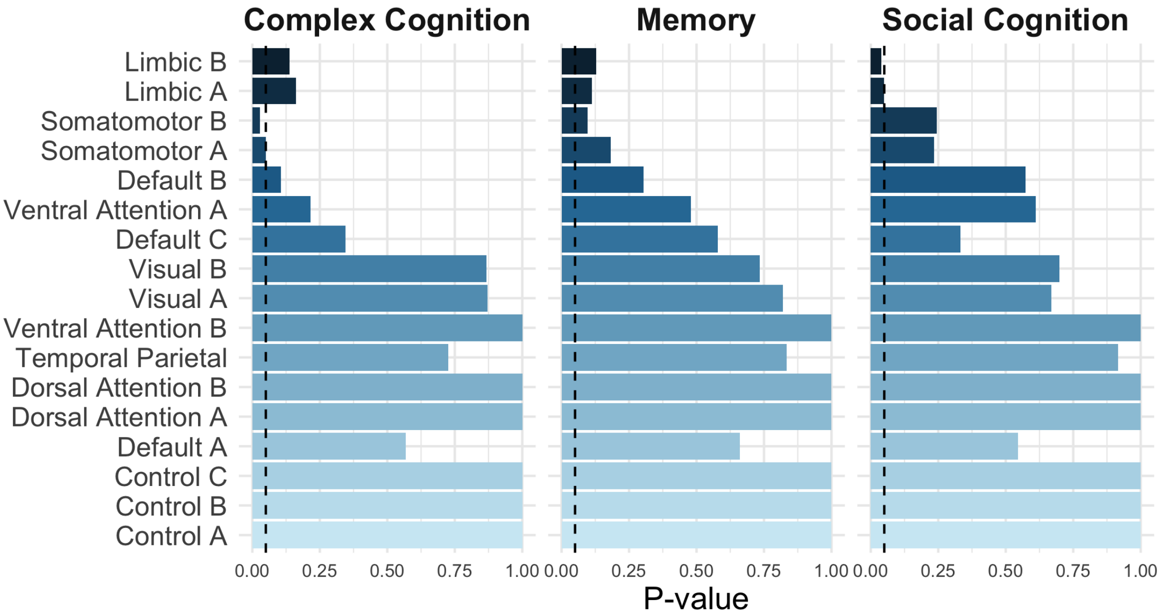

Figure 8: Uncorrected p-values obtained from NETDOM when assessing whether negative associations between activation during an n-back working memory task and neurocognitive scores were enriched in seventeen functional networks. The dashed line represents a $p = .05$ cutoff, yet no networks remained significant after applying a false discovery rate correction.

B.3 Enrichment Tests with Yeo 7-Network Partition – ABCD

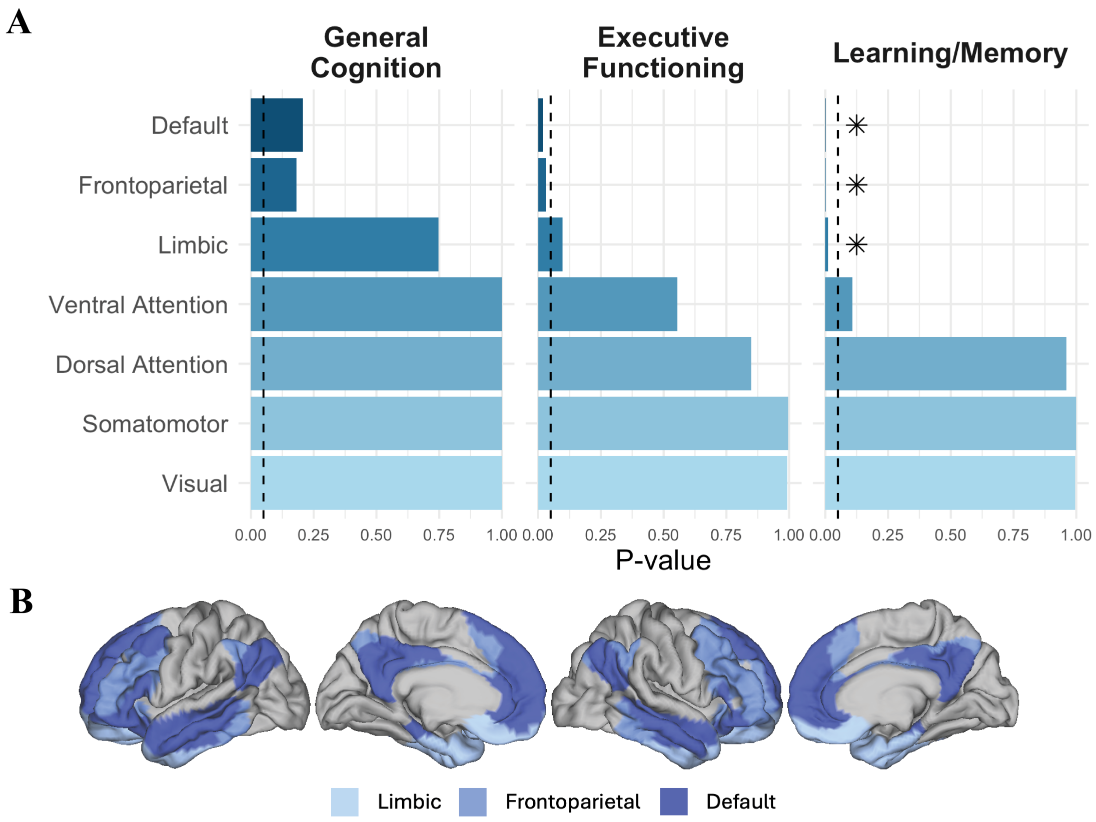

Figure 9: (A) Uncorrected p-values from NETDOM when assessing whether negative associations between cortical thickness and neurocognitive scores were enriched in seven functional networks (Yeo et al., 2011). Stars indicate networks where enrichment tests remained significant after a false discovery rate correction. (B) Functional networks where enriched associations were detected.

B.4 Enrichment Tests with Yeo 7-Network Partition – PNC 1168

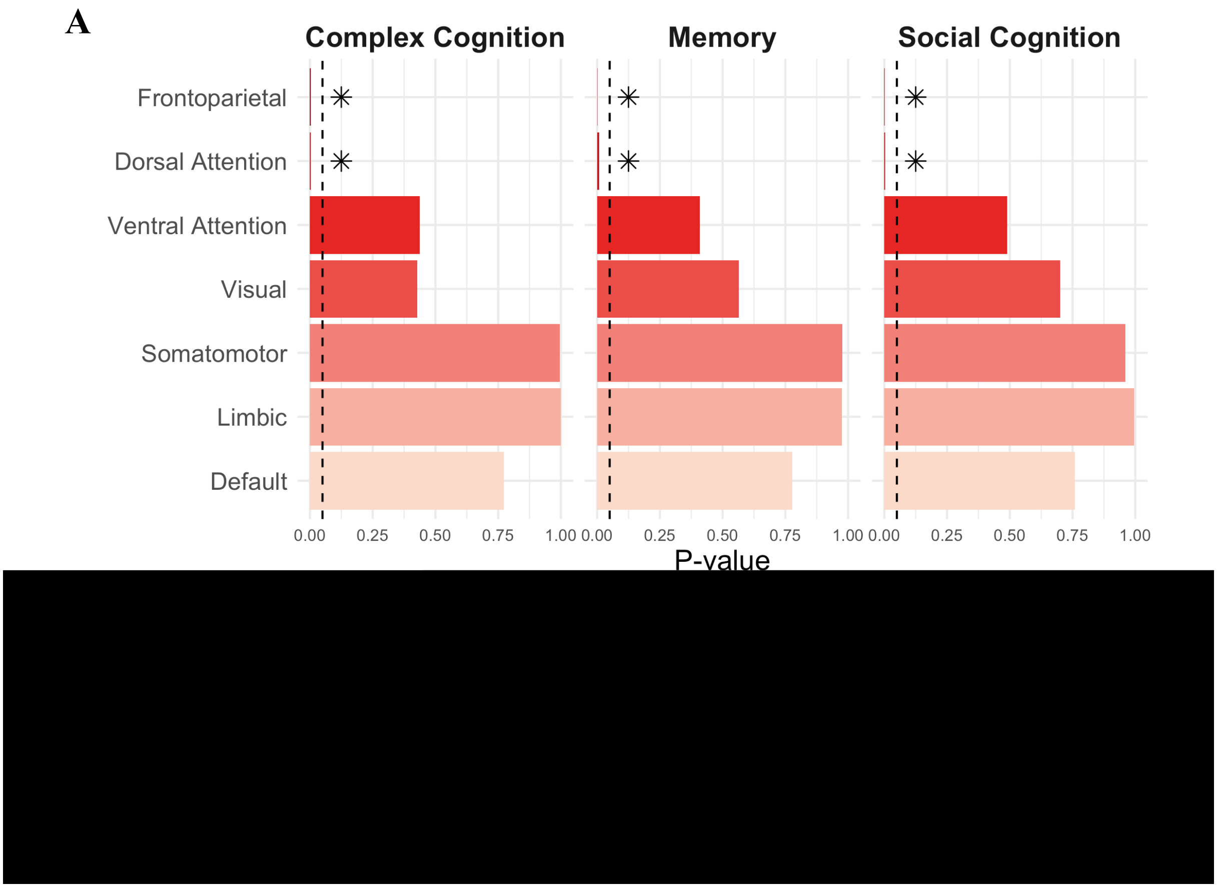

[redacted]

Figure 10: (A) Uncorrected p-values from NETDOM when assessing whether positive associations between activation during a working memory n-back task and neurocognitive scores were enriched in seven functional networks (Yeo et al., 2011). Stars indicate networks where enrichment tests remained significant after a false discovery rate correction. (B) Functional networks where enriched associations were detected.

B.5 Computation Time

	Spatial Resolution			
N	$V = 1000$	$V = 5000$	$V = 10000$	$V = 18715$
50	9.9 (9.87 – 10.2)	39.2 (39 – 39.9)	76.4 (75.5 – 78.1)	139 (138 – 141)
100	12.8 (12.7 – 13.1)	55.6 (55.3 – 56.3)	107 (107 – 108)	196 (195 – 198)
200	17.9 (17.8 – 18.4)	85.2 (84.4 – 86.4)	169 (168 – 170)	304 (300 – 308)
300	25.5 (25.3 – 25.7)	119 (118 – 120)	234 (232 – 234)	448 (440 – 468)

Table 1: Runtime (in seconds) of NETDOM when performing an enrichment test within the visual network, defined using a previously established seven network functional atlas (Yeo et al., 2011). To assess scalability with respect to sample size and image resolution, we subsampled participants from the n-back PNC dataset ($N \in 50, 100, 200, 300$) and downsampled images to multiple spatial resolutions ($V \in 1000, 5000, 10000, 18715$). All runtimes were measured using a single computing core; however, runtime can be further reduced on multicore systems by parallelizing the generation of brain–behavior association maps across permuted samples. Reported values correspond to the median (lower quartile – upper quartile) of NETDOM runtime computed over 10 repeated enrichment tests.

B.6 Enrichment Tests from Competing Methods – ABCD

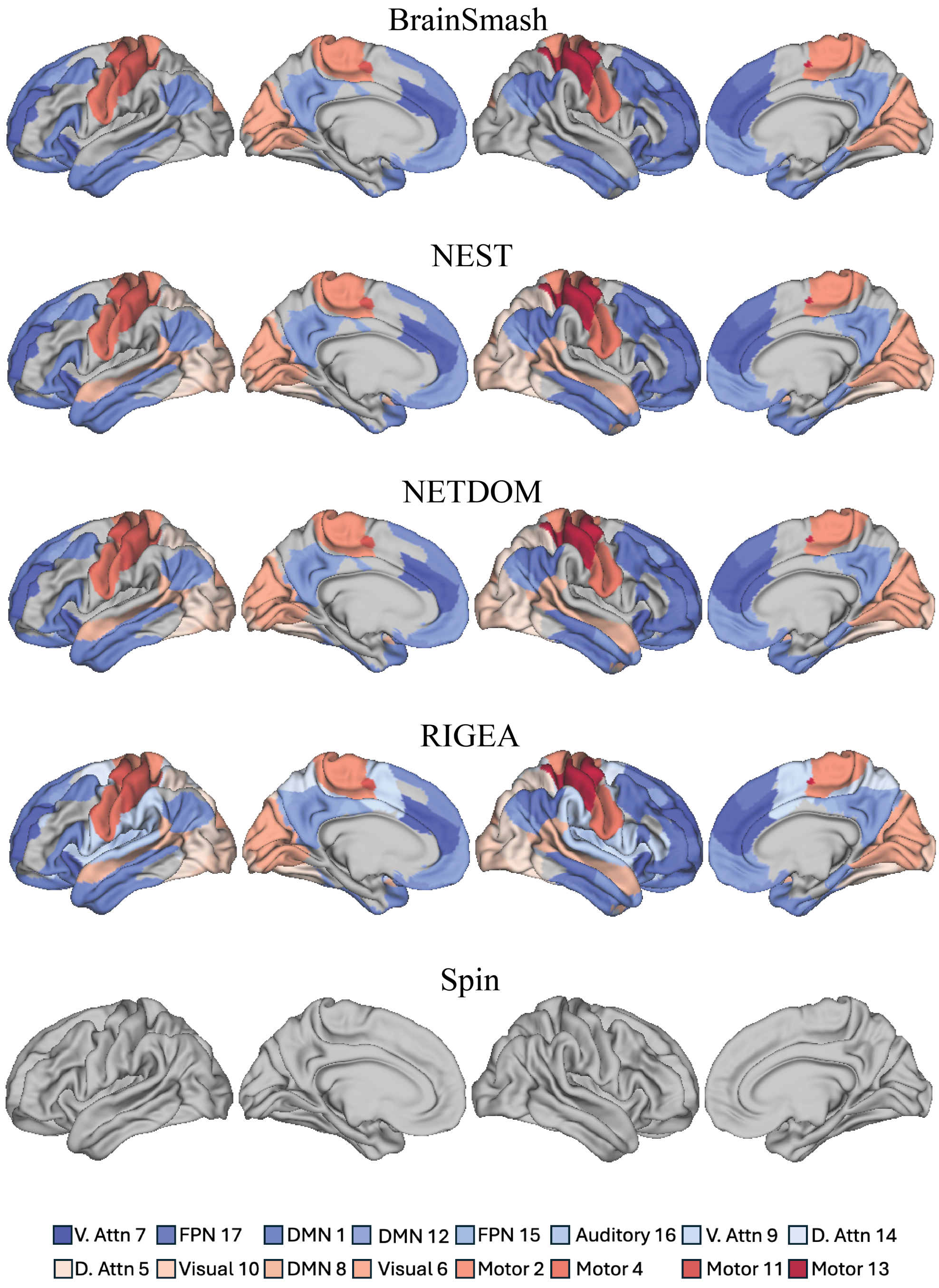

Figure 11: Functional networks where enriched associations between cortical thickness and neurocognitive scores (general cognition, executive functioning, or learning/memory) were detected, with model parameters identical to Figure 5 and Figure 7. Each panel corresponds with a unique method for performing network enrichment tests as described in Section 2.2.3. The results of network enrichment tests in the positive and negative direction were combined into a single figure, such that red colors correspond to networks where positive associations were elevated while blue networks denote regions where negative associations were differentially strong.

B.7 Enrichment Tests from Competing Methods – PNC

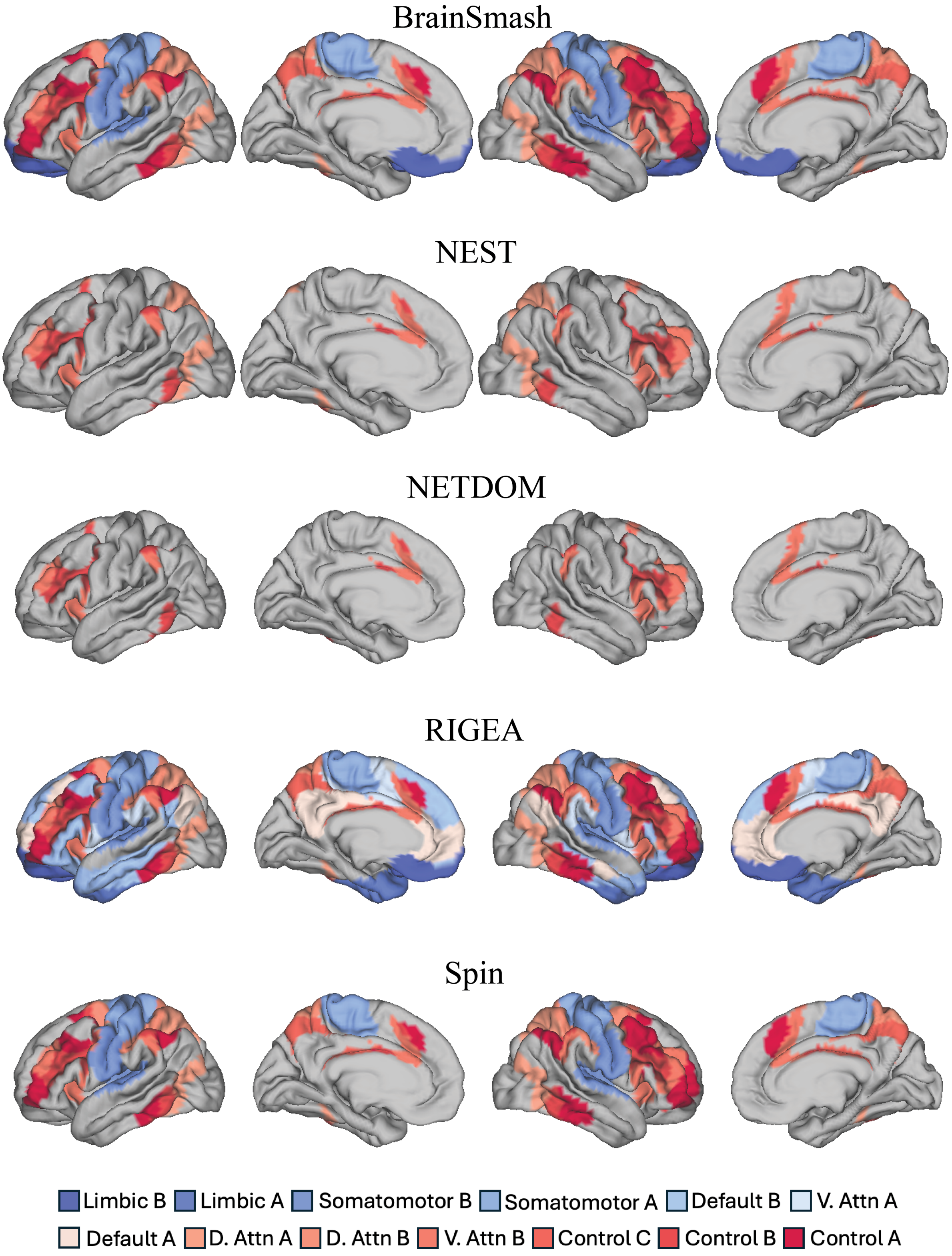

Figure 12: Functional networks where associations between activation during a n-back working memory task and neurocognitive scores (complex cognition, memory, or social cognition) were enriched, with model parameters identical to Figure 6. Each panel corresponds with a unique method for performing network enrichment tests as described in Section 2.2.3. Red and blue colors denote regions where positive and negative associations were enriched, respectively.

B.8 Optimal Tuning Parameter Selected – ABCD

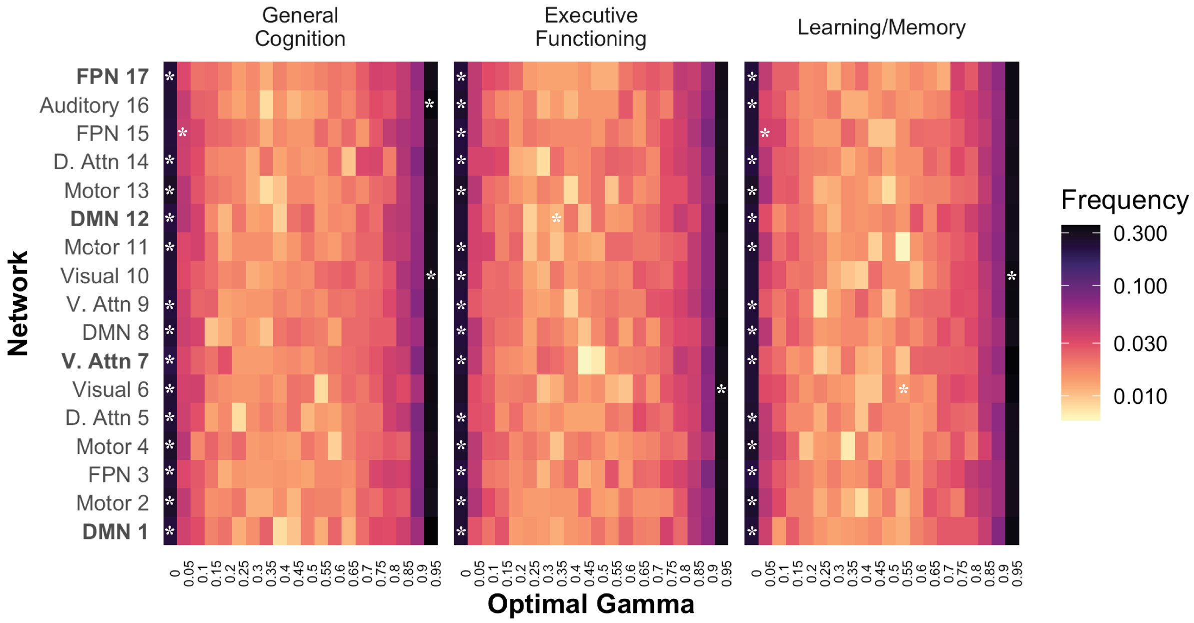

Figure 13: Heatmap representing the distribution of optimal gamma values selected by NETDOM across K = 1000 permuted maps for each of seventeen functional networks and three neurocognitive scores used to test for enrichment of negative thickness-neurocognition associations. Stars indicate the optimal gamma value selected in the observed map, while bold text denotes regions where NETDOM detected enriched associations.

B.9 Optimal Tuning Parameter Selected – PNC

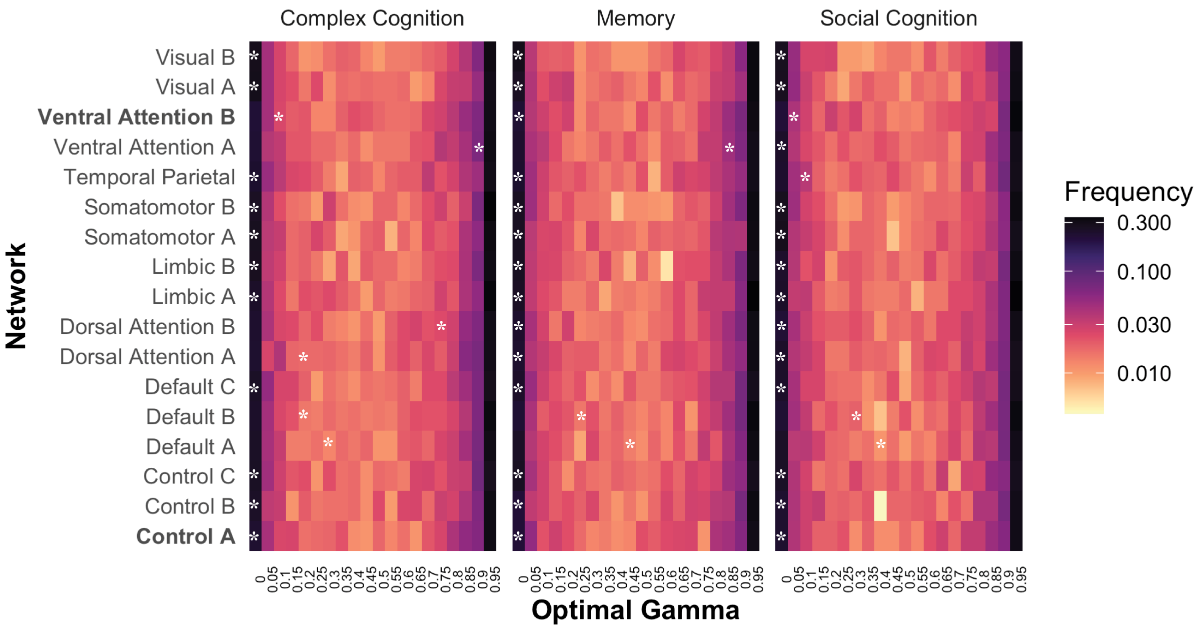

Figure 14: Heatmap depicting the distribution of optimal gamma values selected by NETDOM across K = 1000 permuted maps for each of seventeen functional networks and three neurocognitive scores used to test for network enrichment. Model parameters used to perform enrichment tests were identical to Figure 6. Stars indicate the optimal gamma value selected in the observed map, while bold text denotes regions where NETDOM detected enriched associations.
